# Identification of genomic regions that exhibit sexual dimorphism for size and muscularity in cattle

**DOI:** 10.1093/jas/skab070

**Published:** 2021-03-02

**Authors:** Jennifer L Doyle, Deirdre C Purfield, Tom Moore, Tara R Carthy, Siobhan W Walsh, Roel F Veerkamp, Ross D Evans, Donagh P Berry

**Affiliations:** Teagasc, Animal and Grassland Research and Innovation Centre, Moorepark, Fermoy, Co. Cork, Ireland; Department of Science, Waterford Institute of Technology, Cork Road, Co. Waterford, Ireland; Department of Biological Sciences, Cork Institute of Technology, Bishopstown, Co. Cork, Ireland; School of Biochemistry and Cell Biology, University College Cork, Western Gateway Building, Western Road, Cork, Ireland; Teagasc, Animal and Grassland Research and Innovation Centre, Moorepark, Fermoy, Co. Cork, Ireland; Department of Science, Waterford Institute of Technology, Cork Road, Co. Waterford, Ireland; Animal Breeding and Genomics Centre, Wageningen University and Research Centre, Livestock Research, Wageningen, the Netherlands; Irish Cattle Breeding Federation, Bandon, Co. Cork, Ireland; Teagasc, Animal and Grassland Research and Innovation Centre, Moorepark, Fermoy, Co. Cork, Ireland

**Keywords:** beef cattle, genomics, GWAS, sexual antagonism, sexual dimorphism

## Abstract

Sexual dimorphism, the phenomenon whereby males and females of the same species are distinctive in some aspect of appearance or size, has previously been documented in cattle for traits such as growth rate and carcass merit using a quantitative genetics approach. No previous study in cattle has attempted to document sexual dimorphism at a genome level; therefore, the objective of the present study was to determine whether genomic regions associated with size and muscularity in cattle exhibited signs of sexual dimorphism. Analyses were undertaken on 10 linear-type traits that describe the muscular and skeletal characteristics of both males and females of five beef cattle breeds: 1,444 Angus (**AA**), 6,433 Charolais (**CH**), 1,129 Hereford, 8,745 Limousin (**LM**), and 1,698 Simmental. Genome-wide association analyses were undertaken using imputed whole-genome sequence data for each sex separately by breed. For each single-nucleotide polymorphism (**SNP**) that was segregating in both sexes, the difference between the allele substitution effect sizes for each sex, in each breed separately, was calculated. Suggestively (*P* ≤ 1 × 10^−5^) sexually dimorphic SNPs that were segregating in both males and females were detected for all traits in all breeds, although the location of these SNPs differed by both trait and breed. Significantly (*P* ≤ 1 × 10^−8^) dimorphic SNPs were detected in just three traits in the AA, seven traits in the CH, and three traits in the LM. The vast majority of all segregating autosomal SNPs (86% in AA to 94% in LM) had the same minor allele in both males and females. Differences (*P* ≤ 0.05) in allele frequencies between the sexes were observed for between 36% (LM) and 66% (AA) of the total autosomal SNPs that were segregating in both sexes. Dimorphic SNPs were located within a number of genes related to muscularity and/or size including the *NAB1*, *COL5A2*, and *IWS1* genes on BTA2 that are located close to, and thought to be co-inherited with, the *MSTN* gene. Overall, sexual dimorphism exists in cattle at the genome level, but it is not consistent by either trait or breed.

## Introduction

Sexual dimorphism is the phenomenon whereby males and females of the same species are distinctive in behavior, size, or appearance (Berns, 2013). This is attributable to the combination of sex-specific genes on sex chromosomes, sex-specific expression of genes, and other regulatory mechanisms that are not yet widely understood (Pointer et al., 2013). Sex-dependent differences have been documented for a whole range of traits in different species ranging from color, ornamentation, mating behavior, and size (McPherson and Chenoweth, 2012; Berns, 2013; van der Heide et al., 2016). An individual’s sex is also known to have an influence on the growth of body tissues and could, therefore, affect carcass composition and weight distribution within the body tissue (Berg and Butterfield, 1976). Sexual size dimorphism is likely to have originated in mammals during evolution due to competition among males for access to females; males would fight one another to gain access to females and the winner, generally the bigger, stronger animal would mate with more females (Kirkpatrick, 1987; Katz, 2008). In selective breeding systems, breeding males are selected on numerous desirable traits and consequently competition for mates has been diminished in domesticated animals. Nonetheless, evidence of sexual dimorphism based on quantitative genetics approaches have been reported for several economically important traits in cattle, including growth rate (Koch and Clark, 1955; Marlowe and Gaines, 1958; van der Heide et al., 2016) and carcass traits (Crews and Kemp, 2001; Bittante et al., 2018).

Linear-type traits describing the muscular and skeletal characteristics of an animal are scored globally in both dairy (Veerkamp and Brotherstone, 1997; Berry et al., 2004) and beef (Mc Hugh et al., 2012; Mazza et al., 2014) cattle. These traits are typically considered as being genetically the same in both males and females; estimated genetic correlations of near unity between the same linear-type trait in different sexes of cattle substantiate this assumption (Doyle et al., 2018). Genetic correlations, however, are a manifestation of the cumulative effect of both linkage and pleiotropy across the entire genome and it is possible that the control of such traits by sex may differ in specific genomic locations. The objective, therefore, of the present study was to determine whether genomic regions associated with size and muscularity in cattle exhibited signs of sexual dimorphism. This knowledge will be useful in informing breeding programs of the potential improvement in accuracy achievable by evaluating males and females separately.

## Materials and methods

Animal Care and Use Committee approval was not obtained for the present study as data were obtained from the existing Irish Cattle Breeding Federation (ICBF) national database (http://www.icbf.com).

### Phenotypic data

Linear-type traits are routinely scored in both registered and commercial beef herds by trained classifiers from the ICBF as part of the Irish national beef breeding program (Mc Hugh et al., 2012; Berry and Evans, 2014). The type traits used in the present study describe the muscular and skeletal development of the animal and include development of the hind quarter (**DHQ**), inner thigh (**DIT**), and loin (**DL**), thigh width (**TW**), wither width (**WOW**), wither height (**WH**), back length (**BL**), hip width (**HW**), and chest width (**CW**) and depth (**CD**). The five muscular traits were scored (Supplementary Table S1) on a scale of 1 (narrow) to 15 (wide), whereas the five skeletal traits (Supplementary Table S1) were scored on a scale of 1 (short or narrow) to 10 (long/tall or wide). Data on these 10 traits were available on 147,704 purebred Angus (**AA**), Charolais (**CH**), Hereford (**HE**), Limousin (**LM**), and Simmental (**SI**) beef cattle scored between 6 and 16 mo of age between the years 2000 and 2016.

Data editing procedures and the justification for such edits are outlined in detail by Doyle et al. (2019, 2020a, b). Animals were discarded from the data set if the sire, dam, herd, or classifier was unknown, or the parity of the dam was not recorded. Parity of the dam was subsequently recoded into 1, 2, 3, 4, and ≥5. Contemporary group was defined as herd-by-scoring date generated separately within each breed; each contemporary group had to have at least five records. Each of the 10 traits were separately standardized to a common variance within classifier-by-year as described in detail by Brotherstone (1994). Following edits, data were available on 81,200 animals (Supplementary Table S2) consisting of 3,356 AA, 31,049 CH, 3,004 HE, 35,159 LM, and 8,632 SI.

### Generation of adjusted phenotypes

Prior to inclusion in the genome-wide association analysis, all phenotypes were adjusted within breed in ASREML (Gilmour et al., 2009) using the model:


$$\begin{array}{l} y_{ijkl}=\mu +\;HSD_{i}+\;AM_{j}+\;DP_{k}+\;Animal_{l}+\;e_{ijkl} \end{array}$$

where y_*ijkl*_ is the linear-type trait, μ is the overall mean, HSD_*i*_ is the fixed effect of herd-by-scoring date (*i* = 11,130 levels), AM_*j*_ is the fixed effect of the age in months of the animal (*j* = 11 classes from 6 to 16 mo), DP_k_ is the fixed effect of the parity of the dam (*k* = 1, 2, 3, 4, and ≥5), Animal_*l*_ is the random additive genetic effect of the animal where $N(0,A\sigma_{a}^{2})$, and e is the random residual effect where $N(0,A\sigma_{e}^{2})$; $\sigma_{a}^{2}\;$ is the additive genetic variance, $\sigma_{e}^{2\;}$ is the residual variance, **A** is the numerator relationship matrix, and **I** is an identity matrix. The adjusted phenotype used in the subsequent analysis was the raw phenotype less the fixed-effect solutions of HSD, AM, and DP. This approach of pre-adjustment of phenotypes was undertaken (as opposed to fitting directly in the association analysis) so as to generate a better estimate of especially the contemporary group effect that would have contained nongenotyped animals.

### Genotype data

Of the phenotypic data set of 81 200 animals, 19 449 animals from the five beef breeds (Supplementary Table S2) were imputed to whole-genome sequence (**WGS**) as part of a larger data set of 638 662 multi-breed genotyped animals (Purfield et al., 2019). These 638 662 animals were genotyped using one of seven different genotype panels as described previously by Doyle et al. (2020a, b). The reference population used for imputation contained 90% male animals and 8% female animals; 2% of the reference population were of unknown sex. Each animal had to have a call rate ≥ 90% and only single-nucleotide polymorphisms (**SNPs**) with a known chromosome and position on UMD 3.1, and SNPs with a call rate ≥ 90% within the panel were retained for imputation. 

All autosomes of genotyped animals were imputed to WGS following the steps outlined in Doyle et al. (2020a, b). Imputation of the pseudoautosomal region (**PAR**) and non-PAR regions of the X chromosome was undertaken separately. The non-PAR region was imputed for males and females separately. The PAR region of the X chromosome was defined from 143,861,798 to 148,823,899 bp (Mao et al., 2016). Regions of poor WGS imputation accuracy were discarded as described by Purfield et al. (2019). Furthermore, within each breed and each sex, all SNPs with a minor allele frequency (**MAF**) ≤ 0.002 were not considered further (Supplementary Table S2). The number of SNPs remaining for each sex in each breed is outlined in Supplementary Table S2.

### Genome-wide association

Whole-genome association analyses were performed within each sex in each breed separately using a mixed linear model association analysis in GCTA (Yang et al., 2011). Autosomal SNPs from the original high-density (**HD**) panel (i.e., 734,159 SNPs) were used to construct the genomic relationship matrix (**GRM**) for each sex within each breed as per Doyle et al. (2020a, b) who used the data from the present study but in a combined analysis of both sexes. In the association analyses of the X chromosome, all males were coded as homozygous for the genotyped allele for SNPs in the non-PAR region, while heterozygous SNPs were accepted in the PAR region. The model used for the within-sex and within-breed analysis was as follows:


$$\begin{array}{l} y=\mu +xb+u+e \end{array}$$

where **y** is a vector of preadjusted phenotypes, µ is the overall mean, **x** is the vector of imputed genotypes, **b** is the vector of additive fixed effects of the candidate SNP to be tested for association, $u∼\;N(0,G\sigma_{u}^{2})$ is the vector of additive genetic effects, where **G** is the genomic relationship matrix calculated from the HD SNP genotypes, and $\sigma_{u}^{2}$ is the additive genetic variance, and $e∼N(0,I\sigma_{e}^{2})$ is the vector of random residual effects, where **I** is the identity matrix and $\sigma_{e}^{2}$ is the residual variance.

### Dimorphism

For each SNP that was analyzed in both sexes (i.e., segregating in both sexes), the difference between the allele substitution effect sizes for each sex, in each breed separately, was calculated using a *t*-test:


$$\begin{array}{l} t=\left|\frac{b_{m}-b_{f}}{\sqrt{\frac{{SE}_{m}^{2}+{SE}_{f}^{2}}{n_{m}+n_{f}}}}\right| \end{array}$$

where *b*_*x*_ is the allele substitution effect in males (m) and females (f), SE is the estimated standard error of the allele effect, and *n* is the respective sample size. The presence of dimorphism was determined at each SNP based on the calculated *P*-value from the *t*-test statistic. An SNP with a *P*-value ≤ 1 × 10^−5^ was assumed to have a suggestively different allele effect in the two sexes, whereas an SNP with a *P*-value ≤ 1 × 10^−8^ was assumed to have a significantly different allele substitution effect in the two sexes. These levels of significance are generally consistent with recommended levels (Pe’er et al., 2008). Nonetheless, an additional test was undertaken in the CH and LM breeds where two populations were generated: the first population included half the males and half the females combined with the second population consisting of the remainder. Association analyses were undertaken in each population and the allele effects compared statistically to mimic the approach used in the present study to detect dimorphism. The significance of the difference in allele substation effect was not <1 × 10^−8^, thus providing confidence in this threshold used in this study.

### Quantitative trait loci detection

To identify quantitative trait loci (**QTL**) regions that were dimorphic in more than one trait or more than one breed, each chromosome was split into 1 kb genomic windows and windows containing at least one suggestive (*P* ≤ 1 × 10^−5^) or significant (*P* ≤ 1 × 10^−8^) SNP were compared across the traits and breeds.

## Results

The scale of measurement, mean, and standard deviation of the linear-type traits in each sex in each breed is in Supplementary Table S1. Single-nucleotide polymorphisms with evidence of significant (*P* ≤ 1 × 10^−8^) dimorphism were detected for some traits, while suggestively (*P* ≤ 1 × 10^−5^) dimorphic SNP were detected for all of the traits in all five breeds; however, these SNPs differed both by trait and by breed.

### Angus

A total of 16,541,913 SNPs were segregating in the 1,044 males and 15,402,160 SNPs were segregating in the 400 females. Of these totals, 15,008,408 SNPs were segregating in both the male and female populations (Supplementary Table S2). Significant dimorphism (*P* ≤ 1 × 10^−8^) was evident for a total of seven SNPs across just three traits (HW, TW, and DIT; Table 1), whereas suggestive dimorphism (*P* ≤ 1 × 10^−5^) was evident for between 31 (DHQ) and 1,254 (HW) SNPs depending on the trait (Table 1). In general, the allele substitution effects of the dimorphic SNPs tended to be in opposite directions in each sex (i.e., if the allele effect in the male population was negative, then the allele effect of the same allele in the female population was positive or vice versa; Tables 2and 4). The allele effects in the male population also tended to be closer to zero than those in the female population (Table 2 and 4) and the most significantly dimorphic traits tended to have a very low MAF in the female population. Of the muscular traits investigated, the most significantly dimorphic SNP was an intronic SNP (*P* = 3.45 × 10^−9^) located within the *ADGRA3* gene on Bos Taurus autosome 6 (**BTA6**) and was associated with DIT (Table 2); this SNP had an allele effect of +0.12 (SE = 0.13) and an MAF of 0.026 in males but an allele effect of −3.68 (SE = 0.63) and an MAF of 0.003 in females. Of the skeletal traits, the most significantly dimorphic SNP was an intergenic SNP, rs208222963 (*P* = 9.86 × 10^-10^), located on BTA8 that was associated with HW (Table 3); this SNP had an allele substitution effect of −0.23 (SE = 0.10) in males but +2.29 (SE = 0.40) in females with an MAF of 0.025 in the males and 0.005 in the females.

**Table 1.** The number of suggestively dimorphic (*P* ≤ 1 × 10^−5^) and significantly dimorphic (*P* ≤ 1 × 10^−8^; in parenthesis) SNPs for each trait in each breed^1^

Trait^2^	AA	CH	HE	LM	SI
CD	259	1,439 (84)	148	699 (5)	679
CW	259	3,051 (172)	256	1,105 (27)	136
BL	34	176 (1)	241	229	87
HW	1,254 (1)	272	264	107	178
WH	155	62	122	95	115
DHQ	31	341 (28)	82	433 (3)	115
DIT	329 (5)	51	39	241	91
DL	274	101 (9)	97	74	233
TW	128 (1)	216 (9)	62	46	160
WOW	42	125 (9)	67	268	92

^1^AA, Angus; CH, Charolais; HE, Hereford; LM, Limousin; SI, Simmental.^2^CD, chest depth; CW, chest width; BL, back length; HW, hip width; WH, withe height; DHQ, development of hind quarter; DIT development of inner thigh; DL, development of loin; TW, thigh width; WOW, width of withers. Where there is no parenthesis, no significantly dimorphic SNP was detected.

**Table 2.** The location of the most significantly dimorphic SNPs, limited to the top 5, which were associated with the muscular traits in the AA

							Male		Female		
Trait^1^	Chr	Start	End	No. of dimorphic SNPs	Location of most significantly dimorphic SNP	Updated position of most significantly dimorphic SNP (ARS-UCD 1.2)	Allele frequency	Allele effect (SE)	Allele frequency	Allele effect (SE)	Significance of dimorphism
DHQ	10	101,970,186	102,988,802	4	102,482,010^a^	10,142,4768	0.017	−0.25 (0.14)	0.008	1.53 (0.37)	6.64 × 10^−6^
	12	61,322,071	62,452,555	6	61,890,529^a^	61,531,778	0.118	−0.12 (0.06)	0.118	0.41 (0.10)	3.60 × 10^−6^
	14	33,610,209	34,610,573	2	34,110,209^b^	32,047,165	0.122	0.10 (0.06)	0.140	−0.37 (0.09)	5.32 × 10^−6^
	20	15,474,737	16,474,737	1	15,974,737^a^	15,988,960	0.034	−0.41 (0.10)	0.056	0.10 (0.14)	1.30 × 10^−6^
	21	53,862,500	54,867,369	5	54,367,218^a^	53,876,946	0.020	0.39 (0.13)	0.015	−0.90 (0.25)	4.16 × 10^−6^
DIT	5	10,273,554	11,452,988	5	10,773,554^b^	10,714,008	0.004	0.50 (0.31)	0.003	−3.28 (0.63)	7.04 × 10^−8^
	6	42,909,192	44,746,796	7	43,497,285^b^	42,045,392	0.026	0.12 (0.13)	0.003	−3.68 (0.63)	3.45 × 10^−9^
	14	33,610,209	35,958,833	69	34,110,209^b^	32,047,165	0.122	0.12 (0.06	0.140	−0.49 (0.10)	8.22 × 10^−8^
	20	15,474,737	17,005,863	10	15,974,737^a^	15,988,960	0.034	−0.39 (0.12)	0.056	0.58 (0.14)	1.50 × 10^−7^
	26	17,899,058	19,802,025	5	18,537,518^bc^	18,672,598	0.005	0.88 (0.30)	0.003	−4.42 (0.89)	1.44 × 10^−8^
DL	2	101,653,384	102,770,408	4	102,270,408^a^	101,752,093	0.005	−0.45 (0.27)	0.004	2.60 (0.56)	9.12 × 10^−7^
	5	99,020,985	100,028,321	3	99,527,732^a^	99,094,471	0.025	0.30 (0.12)	0.039	−0.70 (0.15)	3.75 × 10^−7^
	6	117,849,749	118,880,939	4	118,349,749^d^	113,543,086	0.007	−0.59 (0.24)	0.004	2.46 (0.55)	4.29 × 10^−7^
	11	76,054,540	77,056,745	11	76,556,745^a^	76,492,699	0.207	0.07 (0.05)	0.133	−0.46 (0.09)	2.36 × 10^−7^
	21	53,940,632	55,017,766	6	54,450,845^a^	53,960,572	0.011	0.40 (0.18)	0.014	−1.28 (0.27)	4.06 × 10^−7^
TW	8	65,764,830	67,442,321	23	66,797,263^a^	66,310,487	0.004	−1.25 (0.31)	0.003	2.78 (0.70)	1.53 × 10^−7^
	9	29,472,999	30,503,566	6	29,977,509^a^	29,598,390	0.039	−0.36 (0.11)	0.086	0.39 (0.12)	5.43 × 10^−6^
	10	54,322,355	55,487,705	8	54,835,748^b^	54,777,779	0.008	−0.66 (0.24)	0.004	2.35 (0.57)	1.21 × 10^−6^
	12	49,908,375	50,963,553	5	50,408,375^a^	50,050,440	0.010	−0.71 (0.22)	0.003	2.78 (0.70)	1.95 × 10^−6^
	24	59,024,887	60,257,758	24	59,524,887^a^	59,018,608	0.008	0.77 (0.23)	0.011	−1.49 (0.30)	3.90 × 10^−9^
WOW	2	34,064,457	35,064,503	2	34,564,503^b^	34,459,316	0.329	0.12 (0.05)	0.386	−0.32 (0.07)	6.09 × 10^−7^
	3	2,519,849	3,522,479	2	3,019,849^a^	2,958,782	0.009	0.69 (0.23)	0.008	−1.71 (0.43)	1.03 × 10^−6^
	9	2,753,165	3,753,801	6	3,253,165^a^	3,188,640	0.015	−0.20 (0.17)	0.011	1.44 (0.31)	3.22 × 10^−6^
	11	76,054,540	77,056,745	11	76,556,745^a^	76,492,699	0.207	0.13 (0.06)	0.133	−0.45 (0.11)	1.27 × 10^−6^
	23	14,054,921	15,055,421	3	14,554,921^a^	14,546,664	0.018	−0.55 (0.17)	0.008	1.58 (0.43)	5.12 × 10^−6^

1DHQ, development of hind quarter; DIT, development of inner thigh; DL, development of loin; TW, thigh width; WOW, width of withers.

^a^Intergenic variant.

^b^Intron variant.

^c^Downstream gene variant.

^d^Upstream gene variant.

**Table 3.** The location of the most significantly dimorphic SNPs, limited to the top 5, which were associated with the skeletal traits in the AA

							Male		Female		
Trait^1^	Chr	Start	End	No. of dimorphic SNPs	Location of most significantly dimorphic SNP (UMD 3.1)	Updated position of most significantly dimorphic SNP (ARS-UCD 1.2)	Allele frequency	Allele effect (SE)	Allele frequency	Allele effect (SE)	Significance of dimorphism
WH	5	110,840,144	111,861,436	4	111,346,952^b^	110,829,906	0.010	−0.25 (0.17)	0.006	1.63 (0.33)	4.3 × 10^−7^
	8	60,455,638	62,004,178	27	61,312,774^a^	60,912,522	0.084	−0.20 (0.06)	0.014	1.07 (0.24)	2.45 × 10^−7^
	10	31,872,810	32,902,266	7	32,402,196^b^	32,316,973	0.091	−0.12 (0.06)	0.036	0.61 (0.14)	7.23 × 10^−7^
	18	6,104,766	71,13,421	4	6,613,421^a^	6,584,541	0.276	0.09 (0.04)	0.01	−0.32 (0.07)	8.69 × 10^−7^
	29	48,236,260	49,251,635	2	48,751,635^a^	48,087,103	0.011	−0.21 (0.16)	0.006	1.63 (0.33)	4.25 × 10^−7^
BL	1	70,204,850	71,422,791	16	70,835,180^b^	70,223,171	0.259	0.11 (0.04)	0.224	−0.30 (0.07)	2.45 × 10^−7^
	2	28,943,486	30,056,216	3	29,556,216^a^	29,476,541	0.009	0.68 (0.18)	0.026	−0.46 (0.17)	3.28 × 10^−6^
	6	26,975,924	27,975,924	1	27,475,924^b^	26,065,467	0.067	−0.11 (0.07)	0.033	0.70 (0.15)	1.19 × 10^−6^
	8	65,886,591	67,412,672	3	66,912,672^a^	66,423,762	0.019	−0.35 (0.13)	0.006	1.46 (0.36)	2.12 × 10^−6^
	23	23,943,976	24,943,976	1	24,443,976^b^	24,699,576	0.008	−0.30 (0.18)	0.006	1.60 (0.35)	1.30 × 10^−6^
HW	1	62,250,123	63,302,205	6	62,787,935^a^	62,183,938	0.023	−0.10 (0.10)	0.009	1.58 (0.30)	9.27 × 10^−8^
	8	57,066,889	67,412,672	77	66,296,263^a^	65,817,557	0.025	−0.23 (0.10)	0.005	2.29 (0.40)	9.86 × 10^−10^
	10	81,021,198	83,021,473	3	81,521,198^c^	81,172,739	0.011	−0.25 (0.14)	0.003	2.84 (0.55)	5.48 × 10^−8^
	11	30,092,116	31,173,851	16	30,670,702^d^	30,824,114	0.022	−0.20 (0.12)	0.003	2.84 (0.55)	6.34 × 10^−8^
	13	6,530,211	7,544,606	3	7,030,211^b^	6,887,666	0.007	−0.46 (0.19)	0.003	2.87 (0.56)	1.72 × 10^−8^
CW	2	12,527,638	14,665,457	29	16,317,185^a^	16,287,817	0.008	0.48 (0.18)	0.003	−2.24 (0.52)	6.46 × 10^−7^
	6	42,435,811	43,438,281	2	42,938,281^b^	41,487,714	0.497	−0.05 (0.03)	0.424	0.27 (0.06)	9.43 × 10^−7^
	6	46,088,094	47,172,011	47	46,597,538^a^	45,051,418	0.200	−0.10 (0.04)	0.140	0.32 (0.08)	1.08 × 10^−6^
	15	74,696,711	75,749,423	10	75,205,777^b^	74,292,869	0.085	−0.07 (0.06)	0.034	0.75 (0.15)	2.27 × 10^−7^
	28	23,985,323	24,991,309	3	24,485,323^a^	24,334,640	0.080	0.11 (0.06)	0.068	−0.47 (0.10)	4.54 × 10^−7^
CD	3	87,713,085	88,819,263	2	88,213,085^a^	87,638,290	0.018	−0.29 (0.12)	0.015	0.82 (0.19)	8.77 × 10^−7^
	6	76,026,228	77,061,527	89	76,542,923^a^	74,889,187	0.287	−0.10 (0.03)	0.228	0.22 (0.06)	7.15 × 10^−7^
	11	48,278,616	49,764,300	31	48,778,616^a^	48,908,041	0.165	0.11 (0.04)	0.196	−0.26 (0.06)	2.11 × 10^−7^
	23	3,078,545	4,088,215	44	3,581,168^b^	3,660,670	0.044	−0.13 (0.07)	0.008	1.28 (0.28)	9.81 × 10^−7^
	27	16,617,773	17,634,805	3	17,128,718^a^	18,055,239	0.499	0.09 (0.03)	0.433	−0.17 (0.05)	1.21 × 10^−6^

1WH, wither height; BL, back length; HW, hip width; CW, chest width; CD, chest depth.

^a^Intergenic variant.

^b^Intron variant.

^c^Missense variant.

^d^3′ UTR variant.

**Table 4.** The location of the most significantly dimorphic SNPs, limited to the top 5, which were associated with the muscular traits in the CH

							Male		Female		
Trait^1^	Chr	Start	End	No. of dimorphic SNPs	Most significantly dimorphic SNP	Updated position of most significantly dimorphic SNP (ARS-UCD 1.2)	Allele frequency	Allele effect (SE)	Allele frequency	Allele effect (SE)	Significance of dimorphism
DHQ	1	103,824,917	105,170,930	25	104,363,453^a^	103,548,640	0.215	−0.04 (0.03)	0.208	0.21 (0.04)	3.86 × 10^−7^
	2	1,255,471	9,445,764	113	5,302,042^a^	5,369,735	0.497	−0.21 (0.02)	0.496	0.12 (0.03)	1.55 × 10^−15^
	12	45,346,059	48,106,494	85	47,588,546^a^	47,283,052	0.107	−0.08 (0.03)	0.085	0.25 (0.06)	6.02 × 10^−7^
	15	60,710,700	61,831,852	4	61,210,700^a^	60,427,864	0.005	0.60 (0.16)	0.003	−0.89 (0.26)	1.29 × 10^−6^
	16	70,153,655	71,221,659	3	70,653,655^b^	69,141,596	0.004	−0.45 (0.17)	0.004	1.05 (0.23)	1.45 × 10^−7^
DIT	1	117,119,798	118,119,961	2	117,619,798^b^	116,726,201	0.204	0.47 (0.14)	0.219	−0.86 (0.23)	7.34 × 10^−7^
	2	37,440,689	38,440,693	2	37,940,693^a^	37,839,039	0.033	0.69 (0.32)	0.038	−2.01 (0.46)	1.46 × 10^−6^
	19	8,575,554	9,576,597	2	9,076,597^a^	8,844,572	0.482	0.35 (0.13)	0.492	−0.80 (0.20)	1.46 × 10^−6^
	26	45,221,800	46,222,229	4	45,721,883^b^	45,385,940	0.486	0.18 (0.12)	0.462	−0.94 (0.19)	8.10 × 10^−7^
	X	107,228,339	108,242,126	8	107,728,339^a^	102,268,740	0.499	0.18 (0.08)	0.498	−0.80 (0.20)	6.48 × 10^−6^
DL	1	84,531,371	85,539,275	3	85,034,032^a^	84,419,934	0.003	−0.12 (0.19)	0.003	1.75 (0.31)	2.74 × 10^−7^
	2	0	648,674	11	148,674^a^	225,688	0.482	−0.08 (0.03)	0.470	0.14 (0.04)	4.37 × 10^−7^
	2	4,801,997	6,104,335	12	5,587,046^b^	5,654,486	0.487	0.15 (0.03)	0.467	−0.13 (0.04)	1.36 × 10^−10^
	3	51,686,577	52,703,486	2	52,186,577^a^	52,028,651	0.020	−0.09 (0.08)	0.022	0.56 (0.11)	1.33 × 10^−6^
	15	26,121,639	27,171,054	3	26,671,054^b^	26,249,705	0.034	−0.16 (0.07)	0.017	0.60 (0.13)	3.89 × 10^−7^
TW	2	4,801,997	6,104,335	13	5,587,046^b^	5,654,486	0.487	0.17 (0.03)	0.467	−0.16 (0.04)	8.62 × 10^−13^
	5	51.522,594	52,583,478	5	52,022,594^b^	51,784,070	0.016	0.15 (0.10)	0.012	−0.78 (0.16)	8.37 × 10^−7^
	6	100.685.822	102,462,671	3	101,962,671^a^	100,182,435	0.236	−0.12 (0.03)	0.250	0.12 (0.04)	1.64 × 10^−6^
	21	37.454.121	39,897,176	100	37,969,433^a^	37,571,983	0.420	0.05 (0.03)	0.425	−0.17 (0.04)	8.68 × 10^−7^
	27	39,790,937	40,817,098	7	40,317,098^a^	40,462,706	0.295	−0.06 (0.03)	0.320	0.17 (0.04)	9.75 × 10^−7^
WOW	1	25,066,241	26,077,136	3	25,577,136^a^	26,076,126	0.095	0.09 (0.04)	0.126	−0.24 (0.05)	4.07 × 10^−7^
	2	5,064,592	6,104,335	9	5,587,046^b^	5,654,486	0.487	0.15 (0.03)	0.467	−0.12 (0.04)	1.66 × 10^−9^
	4	26,737,341	27,961,476	2	27,461,476^b^	27,487,938	0.002	−1.17 (0.25)	0.003	0.73 (0.29)	5.38 × 10^−7^
	9	60,485,268	61,503,827	4	60,993,475^a^	60,117,188	0.005	−0.73 (0.16)	0.003	1.04 (0.32)	9.89 × 10^−7^
	21	39,307,646	40,307,667	4	39,807,694^a^	39,393,236	0.006	0.37 (0.15)	0.008	−0.80 (0.18)	7.25 × 10^−7^

1DHQ, development of hind quarter; DIT, development of inner thigh; DL, development of loin; TW, thigh width; WOW, width of withers.

^a^Intergenic variant.

^b^Intron variant.

^c^Downstream gene variant.

Of the 1-kb windows containing at least one suggestively associated dimorphic SNP, there was little overlap between the muscular and skeletal groups of traits. The only overlap in windows between the muscular and skeletal traits was between HW, DL and WOW (two windows on each of BTA23 located at 14.555 and 51.713 Mb), between HW and TW (one window on BTA8 located at 66.264 Mb), and between HW and DL (two windows on BTA27 at 18.141 and 18.403 Mb). Only one 1-kb genomic window was suggestively associated with three skeletal traits (WH, BL, and HW; Supplementary Figure S1a), and this was located between 66.386 and 66.387 Mb on BTA8, within the *ENSBTAG00000006446* gene. The greatest overlap across traits in AA was between WH and HW where a total of twelve 1 kb windows across five chromosomes suggestively exhibited sexual dimorphism (Supplementary Figure S1a). Similar to the skeletal traits, only one 1 kb window was common to more than two muscular traits (DL, DIT, and TW) and this was located between 59.524 and 59.525 Mb on BTA24 (Supplementary Figure S2a). The largest overlap across all skeletal traits was between DL and TW where seven 1-kb windows exhibited suggestive dimorphism. Minimal overlap was detected among the remaining skeletal traits

### Charolais

A total of 18,054,274 SNPs were segregating in the 4,641 CH males and 17,448,948 SNPs were segregating in the 1,792 CH females. Of these, 17,227,625 SNPs were segregating in both the male and female animals. Evidence of suggestive dimorphism (*P* ≤ 1 × 10^−5^) existed for between 51 (DIT) and 3,051 (CW) SNPs depending on the trait (Table 1), whereas evidence of significant dimorphism (*P* ≤ 1 × 10^−8^) was evident in all but three traits (i.e., DIT, HW, and WH). Of the muscular traits, the most significantly dimorphic SNP was rs110487743 (*P* = 1.36 × 10^−10^), an intronic SNP located within the *NAB1* gene on BTA2 which exhibited dimorphic associations for DL (Table 4). Of the skeletal traits, the most significantly dimorphic SNP was for CW and was an intergenic SNP, rs446294174 (*P* = 4.44 × 10^−16^; Table 5) that had an allele effect of −0.03 (SE = 0.18) in males but −3.34 (SE = 0.36) in females with an MAF of 0.015 in males and 0.008 in females.

**Table 5.** The location of the most significantly dimorphic SNPs, limited to the top 5, which were associated with the skeletal traits in the CH

							Male		Female		
Trait^1^	Chr	Start	End	No. of dimorphic SNPs	Most significantly dimorphic SNP	Updated position of most significantly dimorphic SNP (ARS−UCD 1.2)	Allele frequency	Allele effect (SE)	Allele frequency	Allele effect (SE)	Significance of dimorphism
WH	7	967,167,29	97,718,351	3	97,483,338^b^	94,984,764	0.213	−0.08 (0.02)	0.254	0.09 (0.02)	1.50 × 10^−6^
	10	75,208,550	77,306,612	5	75,708,579^a^	75,395,884	0.029	−0.13 (0.05)	0.028	0.28 (0.07)	5.89 × 10^−6^
	17	34,267,907	35,272,234	15	347,70,503^a^	34,374,526	0.120	−0.09 (0.03)	0.113	0.14 (0.04)	1.64 × 10^−6^
	19	33,223,631	34,225,743	6	33,723,631^b^	33,122,406	0.451	0.03 (0.02)	0.499	−0.11 (0.03)	5.95 × 10^−6^
	29	29,284,867	30,316,330	11	29,807,810^a^	29,436,465	0.246	0.03 (0.02)	0.170	−0.16 (0.03)	1.46 × 10^−6^
BL	1	60,942,796	62,141,649	4	61,529,004^a^	60,977,668	0.020	0.08 (0.06)	0.008	−0.76 (0.14)	5.31 × 10^−8^
	8	65,823,397	67,021,631	17	66,520,586^bc^	66,036,297	0.014	0.24 (0.07)	0.014	−0.39 (0.11)	7.09 × 10^−7^
	11	67,394,139	68,443,814	13	67,936,368^b^	67,962,565	0.039	0.11 (0.04)	0.033	−0.29 (0.07)	1.18 × 10^−6^
	12	66,787,629	68,105,969	3	67,354,072^a^	66,817,243	0.005	0.19 (0.11)	0.003	−1.24 (0.22)	6.64 × 10^−9^
	26	16,741,492	17,746,984	6	17,242,026^b^	17,376,909	0.198	−0.06 (0.02)	0.196	0.14 (0.03)	2.43 × 10^−7^
HW	1	71,113,538	72,230,151	34	71,680,557^a^	71,071,338	0.007	−0.18 (0.09)	0.003	1.09 (0.24)	7.09 × 10^−7^
	1	15,460,935	151,629,392	5	150,960,935^c^	*	0.031	0.11 (0.05)	0.046	−0.29 (0.06)	1.96 × 10^−7^
	7	78,851,675	79,854,739	3	79,352,226^a^	77,071,480	0.027	−0.09 (0.05)	0.013	0.49 (0.11)	9.91 × 10^−7^
	19	41,254,273	42,757,991	23	42,257,563^c^	41,624,383	0.127	−0.09 (0.02)	0.116	0.14 (0.04)	1.32 × 10^−6^
	23	40,292,666	41,337,462	14	40,792,666^d^	41,014,434	0.044	0.13 (0.04)	0.052	−0.21 (0.06)	1.49 × 10^−6^
CW	2	39,826,441	43,069,032	11	42,375,687^d^	*	0.003	0.46 (0.38)	0.002	−5.30 (0.71)	1.01 × 10^−12^
	8	24,830,532	26,713,234	9	29,877,151^a^	29,907,983	0.015	−0.03 (0.18)	0.008	−3.34 (0.36)	4.44 × 10^−16^
	9	21,416,858	24,598,647	19	21,916,858^a^	21,654,494	0.007	0.21 (0.26)	0.003	−4.10 (0.52)	1.66 × 10^−13^
	14	5,222,811	6,612,129	22	6,528,804^a^	5,500,130	0.010	0.50 (0.23)	0.006	−3.14 (0.43)	4.49 × 10^−14^
	28	0	865,080	17	365,080^a^	1,347,377	0.006	0.62 (0.28)	0.003	−4.79 (0.63)	6.66 × 10^−15^
CD	2	78,501,288	79,652,702	7	79,001,288^a^	78,633,046	0.005	0.29 (0.27)	0.002	−3.88 (0.56)	2.50 × 10^−11^
	6	22,501,427	30,700,058	17	29,804,490^a^	28,380,999	0.006	0.17 (0.28)	0.003	−4.13 (0.57)	1.55 × 10^−11^
	17	20,292,176	22,351,653	28	20,871,130^a^	20,556,557	0.016	0.01 (0.18)	0.008	−2.36 (0.30)	1.22 × 10^−11^
	19	5,471,820	6,722,162	11	6,222,162^a^	6,015,594	0.013	0.15 (0.19)	0.008	−2.41 (0.33)	1.32 × 10^−11^
	20	68,770,623	70,729,278	50	70,127,722^a^	*	0.002	0.23 (0.46)	0.002	−5.16 (0.57)	2.42 × 10^−13^

^1^WH, wither height; BL, back length; HW, hip width; CW, chest width; CD, chest depth.

^a^Intergenic variant.

^b^Intron variant.

^c^Downstream gene variant.

^d^Upstream gene variant.

*SNP has not been mapped on the latest build

Of the 1-kb windows containing at least one dimorphic SNP, no windows were shared between the skeletal and muscular trait groups. Despite the lack of overlap between the skeletal and muscular traits in the CH, considerable dimorphism was detected across the muscular traits. Across trait dimorphism was detected in four of the five muscular traits (i.e., DHQ, DL, TW, and WOW; Supplementary Figure S2b) where eight 1-kb windows in common between 5.54 and 5.60 Mb on BTA2 contained a suggestively associated dimorphic SNP; only one gene, *NAB1*, was located within this region. An additional 22 1-kb windows on BTA2 were also deemed to exhibit across trait dimorphism for the muscular traits (Supplementary Figure S2b). Compared with the muscular traits, fewer windows containing a suggestive SNP were common among the skeletal traits. Seven 1-kb windows were common to CW and CD (Supplementary Figure S1b), one window on each of BTA4, BTA5, BTA12, and BTAX, and three windows on BTA13 that contained the *BTBD3* gene. A single window on BTA15 at 72.31 Mb was common to both WH and BL.

### Hereford

A total of 17,241,152 SNPs were segregating in the 727 HE males and 16,494,904 SNPs were segregating in the 402 HE females. Of these, 15,991,751 SNPs were segregating in both the male and female animals. In comparison to the AA and CH, evidence of suggestive dimorphism (*P* ≤ 1 × 10^−5^) was evident for fewer SNPs in all of the traits, ranging from 39 (DIT) to 256 (CW) SNPs depending on the trait; there was no evidence of significant dimorphism (*P* ≤ 1 × 10^−8^; Table 1). Similar to the AA, the allele substitution effect of the dimorphic SNPs in the males tended to be in the opposite direction to the allele effect of the same SNP in the females (Table 6and 8). The most significantly dimorphic SNP in the muscular traits was rs798960299 (*P* = 6.30 × 10^−8^), an intronic variant located within the *NR5A2* gene on BTA16 with an allele effect of +0.42 (SE = 0.13) in males but −0.61 (SE = 0.14) in females (Table 6). Of the skeletal traits, the most significantly dimorphic SNP was rs381085044, an intergenic SNP on BTA1 that had a dimorphic association with CW represented by an allele effect of −0.22 (SE = 0.06) in males but +0.32 (SE = 0.08) in females (Table 7).

**Table 6.** The location of the most significantly dimorphic SNPs, limited to the top 5, which were associated with the muscular traits in the HE

							Male		Female		
Trait^1^	Chr	Start	End	No. ofdimorphic SNPs	Most significantly dimorphic SNP	Updated position of most significantly dimorphic SNP (ARS-UCD 1.2)	Allele frequency	Allele effect (SE)	Allele frequency	Allele effect (SE)	Significance of dimorphism
DHQ	1	47,475,388	48,477,830	9	47,975,875^a^	47,609,095	0.440	−0.14 (0.05)	0.460	0.20 (0.06)	5.34 × 10^−6^
	7	38,157,809	39,157,899	3	38,657,899^a^	37,308,156	0.307	−0.20 (0.05)	0.291	0.17 (0.06)	1.58 × 10^−6^
	12	33,106,286	34,154,759	5	33,654,759b	33,414,723	0.008	−0.84 (0.24)	0.002	1.90 (0.56)	6.14 × 10^−6^
	16	79,654,320	81,344,038	20	80,834,643^b^	78,911,900	0.032	0.42 (0.13)	0.041	−0.61 (0.14)	6.30 × 10^−8^
	29	48,954,403	49,974,227	13	49,471,817^a^	*	0.080	0.29 (0.09)	0.092	−0.31 (0.09)	4.83 × 10^−6^
DIT	1	139,386,569	140,386,569	1	139,886,569^b^	138,390,445	0.131	−0.24 (0.07)	0.098	0.31 (0.09)	3.36 × 10^−6^
	6	104,110,238	105,116,799	3	104,610,238^b^	102,834,429	0.030	0.46 (0.14)	0.021	−0.59 (0.18)	4.31 × 10^−6^
	13	42,113,572	43,125,008	4	42,613,572^a^	42,236,685	0.003	−1.57 (0.41)	0.005	1.22 (0.4)	1.09 × 10^−6^
	19	48,026,847	49,047,485	15	48,526,847^b^	47,877,987	0.078	−0.23 (0.09)	0.095	0.33 (0.09)	6.22 × 10^−6^
	20	45,699,238	467,313,54	6	46,213,822^a^	46,189,379	0.169	−0.18 (0.06)	0.193	0.32 (0.07)	1.21 × 10^−7^
DL	3	89,108,056	90,628,279	9	90,128,279^d^	89,550,331	0.072	0.14 (0.09)	0.040	−0.82 (0.16)	1.76 × 10^−7^
	16	16,872,687	17,885,604	2	17,372,687^a^	16,732,806	0.211	0.19 (0.06)	0.183	−0.29 (0.08)	9.83 × 10^−7^
	21	57,418,522	58,508,022	5	57,972,000^b^	57,385,760	0.018	0.44 (0.18)	0.019	−1.06 (0.23)	2.06 × 10^−7^
	23	44,376,366	45,381,654	9	44,876,460^b^	45,012,825	0.433	−0.18 (0.05)	0.445	0.19 (0.06)	2.30 × 10^−6^
	26	24,760,894	25,969,246	11	25,358,414^d^	25,093,228	0.052	0.06 (0.10)	0.016	−1.32 (0.25)	2.54 × 10^−7^
TW	4	30,981,541	31,996,449	3	31,481,541^a^	31,358,361	0.052	−0.24 (0.11)	0.050	0.61 (0.15)	2.84 × 10^−6^
	11	34,683,559	35,683,570	2	35,183,559^a^	35,342,330	0.210	−0.17 (0.06)	0.249	0.28 (0.07)	1.98 × 10^−6^
	15	48,172,990	49,186,437	10	48,675,268^c^	48,028,112	0.010	−0.59 (0.25)	0.016	1.07 (0.25)	3.00 × 10^−6^
	27	37,804,950	38,804,962	3	38,304,950^a^	*	0.004	−1.24 (0.38)	0.010	1.25 (0.32)	3.96 × 10^−7^
	29	32,341,813	33,341,891	5	32,841,813^a^	32,298,517	0.010	−0.24 (0.26)	0.005	2.33 (0.45)	6.10 × 10^−7^
WOW	1	23,635,565	24,635,601	2	24,135,565^a^	24,617,417	0.044	0.19 (0.12)	0.027	−0.87 (0.20)	6.32 × 10^−6^
	11	88,551,389	89,554,029	5	89,052,894^a^	89,077,280	0.263	−0.11 (0.06)	0.195	0.34 (0.08)	4.63 × 10^−6^
	16	16,859,305	17,861,510	3	17,361,510^a^	16,721,630	0.297	0.21 (0.06)	0.320	−0.22 (0.07)	9.32 × 10^−7^
	23	28,067,269	29,080,639	4	28,580,639^c^	28,787,480	0.023	−0.66 (0.18)	0.035	0.58 (0.17)	7.84 × 10^−7^
	29	3,021,804	40,53,998	21	3,553,943^a^	3,466,707	0.483	0.14 (0.05)	0.469	−0.28 (0.06)	6.82 × 10^−7^

^1^DHQ, development of hind quarter; DIT, development of inner thigh; DL, development of loin; TW, thigh width; WOW, width of withers.

^a^Intergenic variant.

^b^Intron variant.

^c^Downstream gene variant.

^d^Upstream gene variant.

*SNP has not been mapped on the latest build.

**Table 7.** The location of the most significantly dimorphic SNPs, limited to the top 5, which were associated with the skeletal traits in the HE

							Male		Female		
Trait^1^	Chr	Start	End	No. ofdimorphic SNPs	Most significantly dimorphic SNP	Updated position of most significantly dimorphic SNP (ARS-UCD 1.2)	Allele frequency	Allele effect (SE)	Allele frequency	Allele effect (SE)	Significance of dimorphism
WH	1	1,925,823	2,922,535	2	2,422,535^b^	3,142,926	0.010	0.63 (0.20)	0.009	−0.99 (0.27)	1.22 × 10^−6^
	10	49,372,889	50,457,497	10	49,872,889^b^	49,812,963	0.004	−1.27 (0.30)	0.006	0.83 (0.31)	1.18 × 10^−6^
	10	68,414,747	69,451,655	11	68,933,579^b^	68,686,530	0.107	0.27 (0.06)	0.137	−0.20 (0.07)	5.97 × 10^−7^
	16	916,164	1,945,553	12	14,41,711^c^	1,624,685	0.025	−0.36 (0.13)	0.016	0.78 (0.20)	1.05 × 10^−6^
	24	10,954,731	11,969,624	6	11,461,076^a^	11,159,461	0.096	−0.27 (0.06)	0.067	0.29 (0.09)	2.65 × 10^−7^
BL	4	12,086,873	13,087,742	3	12,587,742^a^	12,740,501	0.008	−0.90 (0.24)	0.005	1.22 (0.39)	3.37 × 10^−6^
	8	25,464,831	26,824,440	23	25,966,773^a^	25,943,096	0.110	0.32 (0.07)	0.132	−0.23 (0.08)	3.56 × 10^−7^
	14	30,747,311	31,768,991	12	31,247,311^a^	29,553,248	0.461	0.14 (0.04)	0.471	−0.20 (0.06)	1.38 × 10^−6^
	25	13,061,095	14,195,964	4	13,561,095^b^	*	0.003	−1.27 (0.36)	0.012	0.75 (0.24)	3.63 × 10^−6^
	28	32,468,295	34,072,536	3	33,572,536^b^	33,371,736	0.146	−0.10 (0.06)	0.148	0.37 (0.09)	3.18 × 10^−6^
HW	5	75,173,455	76,244,035	40	75,719,616^a^	75,344,242	0.006	−0.73 (0.28)	0.004	1.85 (0.44)	8.46 × 10^−7^
	11	88,093,610	89,599,089	7	89,005,217^a^	89,030,779	0.263	−0.13 (0.06)	0.195	0.40 (0.09)	8.95 × 10^−7^
	12	82,288,642	83,293,488	3	82,793,488^a^	78,806,868	0.155	−0.17 (0.06)	0.208	0.29 (0.07)	3.07 × 10^−7^
	23	51,480,640	52,527,183	5	52,013,801^b^	52,167,774	0.089	−0.16 (0.08)	0.091	0.46 (0.10)	3.27 × 10^−7^
	27	8,148,686	10,276,462	17	86,48,686^a^	9,657,276	0.107	−0.24 (0.06)	0.106	0.30 (0.09)	5.65 × 10^−7^
CW	1	62,368,829	63,399,292	5	62,868,829^a^	62,264,629	0.082	−0.22 (0.06)	0.108	0.32 (0.08)	5.58 × 10^−8^
	5	49,311,974	50,341,913	23	49,822,014^b^	49,592,445	0.428	−0.17 (0.04)	0.384	0.13 (0.05)	5.20 × 10^−7^
	6	41,424,236	42,580,067	9	42,029,809^b^	40,571,631	0.003	1.31 (0.31)	0.005	−1.13 (0.33)	7.10 × 10^−8^
	9	31,485,678	32,498,136	4	31,985,678^a^	31,570,153	0.010	0.42 (0.19)	0.004	−1.78 (0.38)	2.34 × 10^−7^
	13	2,019,339	3,201,947	16	2,522,257^d^	2,613,918	0.066	0.10 (0.07)	0.030	−0.67 (0.13)	1.94 × 10^−7^
CD	10	2,981,982	4,121,382	3	3,621,377^a^	3,672,531	0.008	0.11 (0.19)	0.002	2.49 (0.48)	3.42 × 10^−6^
	11	67,322,056	68,784,534	4	68,278,582^d^	68,305,651	0.003	−0.87 (0.32)	0.002	2.05 (0.48)	4.15 × 10^−7^
	16	9,638,392	10,667,968	6	10,150,979^a^	95,50,179	0.044	−0.24 (0.08)	0.040	0.46 (0.13)	3.62 × 10^−6^
	19	42,220,630	43,244,434	32	42,738,845^d^	42,096,896	0.349	0.11 (0.03)	0.322	−0.20 (0.06)	2.67 × 10^−6^
	22	25,263,617	26,332,810	15	25,767,332^a^	25,654,951	0.054	−0.15 (0.07)	0.050	0.49 (0.11)	7.27 × 10^−7^

^1^WH, wither height; BL, back lenght; HW, hip width; CW, chest width; CD, chest depth.

^a^Intergenic variant.

^b^Intron variant.

^c^Downstream gene variant.

^d^Upstream gene variant.

*SNP has not been mapped on the latest build.

**Table 8.** The location of the most significantly dimorphic SNPs, limited to the top 5, which were associated with the muscular traits in the LM

							Male		Female		
Trait^1^	Chr	Start	End	No. of dimorphic SNPs	Most significantly dimorphic SNP	Updated position of most significantly dimorphic SNP (ARS-UCD 1.2)	Allele frequency	Allele effect (SE)	Allele frequency	Allele effect (SE)	Significance of dimorphism
DHQ	10	87,342,478	88,342,595	3	87,842,478^a^	86,838,930	0.004	−0.01 (0.15)	0.003	−1.64 (0.23)	1.85 × 10^−9^
	12	88,313,474	89,356,176	25	88,814,730^a^	84,800,697	0.011	0.16 (0.08)	0.005	−0.84 (0.17)	1.96 × 10^−7^
	14	80,691,409	81,691,453	2	81,191,409^a^	78,818,851	0.002	0.10 (0.19)	0.002	−1.46 (0.24)	4.02 × 10^−7^
	16	7,877,931	9,577,924	42	8,703,762^a^	8,116,291	0.003	0.12 (0.16)	0.002	−1.50 (0.24)	2.53 × 10^−8^
	18	53,678,352	54,842,543	90	54,323,356^a^	53,885,291	0.003	0.36 (0.17)	0.002	−1.28 (0.25)	1.03 × 10^−7^
DIT	1	134,576,003	135,674,198	26	135,111,581^a^	*	0.024	−1.21 (0.31)	0.035	1.27 (0.38)	4.64 × 10^−7^
	7	94,624,535	94,624,535	9	95,351,376^a^	92,825,575	0.021	1.27 (0.31)	0.082	−0.92 (0.26)	9.29 × 10^−8^
	8	83,362,119	84,373,117	16	83,862,344^a^	82,440,029	0.026	−0.71 (0.29)	0.029	1.77 (0.43)	2.06 × 10^−6^
	14	12,997,796	14,065,940	91	13,497,889^a^	12,381,763	0.351	−0.39 (0.11)	0.373	0.60 (0.16)	3.15 × 10^−7^
	20	15,771,955	16,909,647	12	16,366,926^a^	16,380,235	0.003	3.16 (0.79)	0.005	−2.93 (0.93)	6.26 × 10^−7^
DL	2	90,388,815	91,414,365	6	90,901,181^a^	90,485,467	0.170	−0.09 (0.03)	0.168	0.12 (0.03)	1.37 × 10^−6^
	6	95,583,095	96,694,369	5	96,194,369^a^	94,427,267	0.004	−0.43 (0.15)	0.002	1.00 (0.27)	4.45 × 10^−6^
	15	81,201,476	82,201,561	2	81,701,476^a^	80,411,671	0.149	0.07 (0.03)	0.171	−0.14 (0.03)	1.04 × 10^−6^
	25	3,582,865	4,590,247	3	4,090,247^bc^	4,072,335	0.492	0.09 (0.02)	0.460	−0.07 (0.03)	5.72 × 10^−7^
	X	33,834,595	34,867,127	35	34,354,746^a^	34,130,068	0.376	−0.03 (0.01)	0.404	0.11 (0.03)	5.35 × 10^−6^
TW	2	19,380,958	20,380,975	3	19,880,958^a^	19,838,410	0.014	0.27 (0.09)	0.011	−0.38 (0.l1)	6.57 × 10^−6^
	2	20,700,909	21,720,973	6	21,217,950^a^	21,181,655	0.100	0.06 (0.03)	0.090	−0.19 (0.05)	7.05 × 10^−6^
	14	6,060,940	7,069,392	3	6,569,392^a^	5,540,717	0.004	−0.36 (0.16)	0.003	0.87 (0.21)	3.45 × 10^−6^
	23	40,690,020	41,776,379	6	41,276,379^b^	41,595,913	0.009	−0.15 (0.10)	0.003	0.94 (0.20)	1.11 × 10− ^6^
	X	16,962,347	17,966,732	2	17,466,732^b^	17,527,360	0.415	−0.02 (0.01)	0.445	0.12 (0.03)	3.38 × 10^−6^
WOW	1	51,070,933	52,117,339	12	51,577,476^a^	51,177,783	0.289	0.07 (0.02)	0.294	−0.10 (0.03)	1.96 × 10^−6^
	2	85,735,779	86,812,555	7	86,265,284^a^	85,863,432	0.019	−0.18 (0.08)	0.032	0.31 (0.07)	2.80 × 10^−6^
	10	28,209,680	29,266,690	175	28,742,392^b^	28,681,684	0.378	−0.07 (0.02)	0.353	0.10 (0.03)	2.53 × 10^−6^
	12	66,608,973	67,613,368	3	67,108,973^b^	66,572,583	0.011	−0.20 (0.10)	0.009	0.60 (0.13)	9.62 × 10^−7^
	19	7,961,956	8,967,691	5	8,466,831^b^	8,237,090	0.003	−0.95 (0.19)	0.002	0.61 (0.27)	1.69 × 10^−6^

^1^DHQ, development of hind quarter; DIT, development of inner thigh; DL, development of loin; TW, thigh width; WOW, width of withers.

^a^Intergenic variant.

^b^Intron variant.

^c^Upstream gene variant.

*SNP has not been mapped on the latest build.

No 1-kb window that contained at least one dimorphic SNP was common to more than two traits. Limited dimorphism was found between CW and HW, where three windows between 40.75 and 40.78 Mb on BTA24 containing the *PTPRM* gene were suggestively associated with both traits (Supplementary Figure S1c). CW also had two separate windows exhibiting dimorphism on BTA13 between 27.46 and 27.47 Mb in common with CD, and one window on BTA14 at 45.485 Mb in common with WH. Two adjacent 1-kb windows on BTA8 at 25.965 Mb were common to both WH and BL. For the muscular traits, WOW had one window in common with each of DL (BTA10 at 48.327 Mb), TW (BTA10 at 50.420 Mb), and DHQ (BTA9 at 83.332 Mb). One 1-kb window was also common to both TW and DHQ (BTA16 at 68.580 Mb; Supplementary Figure S2c).

### Limousin

A total of 18,056,913 SNPs were segregating in the LM males and 17,767,237 SNPs were segregating in the LM females. Of these, 17,482,131 SNPs were segregating in both the male and female animals. Between 46 (TW) and 1105 (CW) SNPs were suggestively dimorphic (*P* ≤ 1 × 10^−5^), whereas three traits (i.e., CW, CD, and DHQ) had evidence of significant dimorphism (*P* ≤ 1 × 10^−8^; Table 1). Of the muscular traits, the most significantly dimorphic SNP was rs42425148 (*P* = 1.85 × 10^−9^), an intergenic SNP located on BTA1 that had a dimorphic association with DHQ (Table 8) represented by an allele effect of −0.01 (SE = 0.15) in males but −1.64 (SE = 0.23) in females. The most significantly dimorphic SNP for the skeletal traits was also an intergenic SNP, rs478688690 (*P* = 3.63 × 10^−11^), located on BTA11, that had a dimorphic association with CW (Table 9), with an allele effect of +0.03 (SE = 0.29) in males and −3.86 (SE = 0.51) in females; this SNP had an MAF of 0.005 in males and 0.003 in females. Similar to the AA and CH, SNPs with a higher MAF tended to have an allele effect that was closer to zero. An intergenic SNP with dimorphic associations in HW (*P* = 5.71 × 10^−7^) had an MAF of 0.493 in males and 0.486 in females, but the allele substitution effects were just −0.07 (SE = 0.01) in males and +0.06 (SE = 0.02) in females.

**Table 9.** The location of the most significantly dimorphic SNPs, limited to the top 5, which were associated with the skeletal traits in the LM

							Male		Female		
Trait^1^	Chr	Start	End	No. of dimorphic SNPs	Most significantly dimorphic SNP	Updated position of most significantly dimorphic SNP (ARS-UCD 1.2)	MAF	Allele effect (SE)	MAF	Allele effect (SE)	Significance of dimorphism
WH	3	9,818,384	10,820,240	2	10,318,384^a^	*	0.022	−0.20 (0.05)	0.013	0.26 (0.08)	1.08 × 10^−6^
	6	36,963,566	40,499,953	40	39,760,256^a^	38,319,979	0.484	−0.07 (0.02)	0.487	0.09 (0.02)	1.07 × 10^−8^
	11	48,105,352	49,267,580	9	48,766,334^a^	48,896,156	0.006	0.26 (0.09)	0.006	−0.49 (0.13)	3.40 × 10^−6^
	19	30,754,934	31,762,424	2	31,254,934^a^	30,619,941	0.257	−0.06 (0.02)	0.248	0.09 (0.02)	1.94 × 10^−7^
	X	16,956,607	17,966,732	6	17,462,347^b^	17,522,975	0.428	−0.01 (0.01)	0.454	0.11 (0.02)	1.19 × 10^−6^
BL	2	19,533,709	20,573,649	21	20,065,919^a^	20,029,900	0.096	−0.06 (0.02)	0.089	0.16 (0.04)	1.02 × 10^−6^
	15	7,167,859	8,174,094	3	7,674,094^b^	7,433,522	0.143	−0.05 (0.02)	0.118	0.13 (0.03)	3.14 × 10^−6^
	16	64,732,311	65,827,907	8	65,324,669^a^	63,842,035	0.003	0.39 (0.14)	0.003	−0.74 (0.19)	1.47 × 10^−6^
	21	10,874,079	11,978,348	2	11,374,079^a^	11,140,251	0.009	−0.12 (0.07)	0.006	0.60 (0.14)	4.03 × 10^−6^
	23	19,728,831	20,739,036	5	20,228,831^b^	20,236,461	0.005	−0.34 (0.10)	0.004	0.55 (0.16)	3.04 × 10^−6^
HW	4	1,742,345	2,747,816	5	2,245,736^a^	2,343,834	0.018	0.15 (0.05)	0.021	−0.27 (0.07)	1.83 × 10^−6^
	6	39,032,482	40,040,409	8	39,539,558^a^	38,099,446	0.493	−0.07 (0.01)	0.486	0.06 (0.02)	5.71 × 10^−7^
	12	5,047,438	6,074,330	3	5,547,438^a^	5,568,301	0.020	−0.14 (0.05)	0.009	0.39 (0.10)	1.23 × 10^−6^
	21	23,430,143	24,464,331	28	23,931,207^a^	23,468,504	0.237	−0.01 (0.02)	0.202	0.11 (0.03)	1.74 × 10^−7^
	29	13,364,114	14,373,070	4	13,872,420^a^	13,797,973	0.011	0.21 (0.07)	0.011	−0.34 (0.09)	1.11 × 10^−6^
CW	11	15,939,177	16,959,054	3	16,459,054^a^	16,439,125	0.005	0.03 (0.29)	0.003	−3.86 (0.51)	3.63 × 10^−11^
	20	60,692,205	68,001,633	7	62,047,530^a^	61,941,499	0.008	0.03 (0.22)	0.002	−3.67 (0.58)	2.64 × 10^−9^
	22	55,794,366	59,621,588	10	56,329,073^a^	55,689,684	0.031	0.31 (0.12)	0.017	−1.26 (0.22)	2.01 × 10^−10^
	26	38,382,849	39,632,022	5	38,919,539^a^	*	0.006	0.40 (0.26)	0.002	−3.51 (0.58)	8.26 × 10^−10^
	X	15,184,800	16,185,025	2	15,684,800^a^	15,786,355	0.004	0.36 (0.21)	0.003	−3.37 (0.53)	6.15 × 10^−11^
CD	3	26,560,340	27,936,092	8	27,203,740^a^	*	0.003	0.59 (0.39)	0.002	−3.35 (0.58)	1.52 × 10^−8^
	6	65,184,840	66,194,780	2	65,684,840^a^	64,045,311	0.003	0.54 (0.39)	0.002	−4.10 (0.65)	8.70 × 10^−10^
	10	66,552,011	70,565,607	71	70,065,607^c^	69,820,365	0.005	0.55 (0.30)	0.002	−3.16 (0.58)	1.69 × 10^−8^
	17	24,086,084	25,381,796	56	24,844,625^a^	*	0.003	0.42 (0.26)	0.002	−2.21 (0.39)	1.95 × 10^−8^
	20	60,681,167	62,562,427	6	61,192,205^a^	61,100,715	0.004	0.21 (0.35)	0.002	−3.81 (0.60)	9.40 × 10^−9^

^1^WH, wither height; BL, back length; HW, hip width; CW, chest width; CD, chest depth.

^a^Intergenic variant.

^b^Intron variant.

^c^Downstream gene variant.

*SNP has not been mapped on the latest build.

Genomic regions that exhibited sexual dimorphism across traits in the LM were limited; eight 1-kb windows were found to be suggestively associated with three of the skeletal traits (WH, BL, HW; Supplementary Figure S1d), whereas only three windows were common between TW and WOW of the muscular traits (Supplementary Figure S2d). All eight windows associated with WH, BL, and HW were on BTA6, but no obvious candidate gene was located in the vicinity. The 40 windows suggestively associated with both CW and CD (Supplementary Figure S1d) were located on 15 different autosomes: BTA5, BTA6, BTA8, BTA9, BTA10, BTA11, BTA13, BTA16, BTA17, BTA18, BTA20, BTA22, BTA24, BTA26, BTA29, and BTAX.

### Simmental

A total of 18,257,175 SNPs were segregating in the SI males, and 17,814,297 SNPs were segregating in the SI females, whereas 17,319,250 of these SNPs were segregating in both the males and females. Between 87 (BL) to 679 (CD) SNPs were suggestively dimorphic (*P* ≤ 1 × 10^−5^), whereas no SNP was significantly dimorphic (*P* ≤ 1 × 10^−8^; Table 1). Once again, the most significantly dimorphic SNPs tended to have a low MAF and a large allele effect size. The most significantly dimorphic SNP associated with any of the muscular traits was rs110995439 (*P* = 1.40 × 10^−8^), an intron variant located within the *GPC5* gene on BTA12 that had a dimorphic association with DIT; the allele substitution effect in the males was −0.85 (SE = 0.34), whereas the allele substitution effect in the females was +1.76 (SE = 0.30; Table 10). Of the skeletal traits, the most significantly dimorphic SNP was an intergenic SNP, rs437227524 (*P* = 1.98 × 10^−8^), that had a dimorphic association with CD (Table 11) and had an allele substitution effect of −0.08 (SE = 0.24) in the male population but −2.64 (SE = 0.42) in the female population with an MAF of 0.004 in the males and 0.002 in the females. An intronic SNP, rs133629874 (*P* = 2.20 × 10^−7^), located within the *MMRN1* that had a dimorphic association with CW had an MAF of 0.439 in males and 0.472 in females with an effect size of +0.14 (SE = 0.03) in males and −0.13 (SE = 0.04) in females.

**Table 10.** The location of the most significantly dimorphic SNPs, limited to the top 5, which were associated with the muscular traits in the SI

							Male		Female		
Trait^1^	Chr	Start	End	No. of dimorphic SNPs	Most significantly dimorphic SNP	Updated position of most significantly dimorphic SNP (ARS-UCD 1.2)	Allele frequency	Allele effect (SE)	Allele frequency	Allele effect (SE)	Significance of dimorphism
DHQ	1	61,306,996	70,287,318	7	61,948,489^a^	61,358,095	0.014	0.63 (0.20)	0.013	−0.75 (0.21)	1.46 × 10^−6^
	16	25,091,200	26,156,103	7	25,645,958^a^	*	0.007	−0.81 (0.28)	0.008	1.12 (0.25)	2.47 × 10^−7^
	18	38,816,641	40,478,326	7	39,974,706^a^	39,838,731	0.003	1.27 (0.40)	0.005	−1.25 (0.32)	1.05 × 10^−6^
	19	48,915,944	50,012,600	4	49,415,944^a^	48,765,663	0.011	−0.40 (0.23)	0.003	2.08 (0.47)	1.86 × 10^−6^
	20	12,847,020	13,908,908	29	13,347,020^b^	13,394,150	0.006	0.99 (0.30)	0.005	−1.10 (0.32)	1.50 × 10^−6^
DIT	6	106,083,805	107,083,825	2	106,583,805^a^	114,749,586	0.002	2.31 (0.55)	0.004	−0.98 (0.38)	8.40 × 10^−7^
	8	13,016,740	14,043,577	6	13,541,906^b^	13,620,555	0.051	0.51 (0.12)	0.082	−0.22 (0.09)	7.91 × 10^−7^
	12	65,403,646	67,717,725	14	67,209,065^a^	66,672,413	0.007	−0.85 (0.34)	0.006	1.76 (0.30)	1.40 × 10^−8^
	17	29,020,755	30,121,482	5	29,520,755^b^	29,099,068	0.008	−1.06 (0.30)	0.003	1.73 (0.48)	7.74 × 10^−7^
	22	2,823,206	3,890,106	3	3,390,106^c^	33,463,86	0.006	−1.65 (0.34)	0.005	0.72 (0.31)	3.22 × 10^−7^
DL	2	127,861,842	128,864,558	2	128,364,558^a^	127,768,904	0.025	−0.23 (0.16)	0.013	1.14 (0.22)	7.57 × 10^−7^
	10	89,463,351	90,472,920	2	89,963,351^c^	88,893,256	0.006	1.09 (0.30)	0.005	−1.23 (0.36)	7.37 × 10^−7^
	12	84,618,243	85,621,166	2	85,118,243^a^	81,125,408	0.087	−0.45 (0.10)	0.061	0.28 (0.11)	4.18 × 10^−7^
	25	35,252,984	36,256,151	4	35,752,984^a^	35,196,894	0.078	−0.27 (0.10)	0.068	0.43 (0.10)	9.22 × 10^−7^
	28	33,866,54	4,391,033	2	3,886,654^a^	3,541,484	0.003	−2.29 (0.50)	0.005	1.06 (0.36)	6.03 × 10^−8^
TW	1	131,926,057	133,026,755	19	132,524,816^a^	131,446,846	0.186	0.23 (0.07)	0.173	−0.24 (0.07)	7.78 × 10^−7^
	2	55,252,366	56,331,341	6	55,802,026^a^	55,583,385	0.022	0.40 (0.19)	0.013	−0.96 (0.23)	3.25 × 10^−6^
	2	99,511,847	100,585,115	3	100,011,847^a^	99,572,436	0.014	1.00 (0.23)	0.013	−0.52 (0.21)	1.23 × 10^−6^
	14	68,627,562	70,444,960	30	69,137,624^a^	66,845,331	0.059	−0.46 (0.11)	0.037	0.32 (0.13)	2.92 × 10^−6^
	18	3,288,714	4,902,994	7	3,802,911^a^	3,762,701	0.015	0.93 (0.22)	0.026	−0.35 (0.16)	2.56 × 10^−6^
WOW	3	97,729,943	99,024,542	2	98,229,943^a^	*	0.015	1.04 (0.24)	0.017	−0.50 (0.22)	2.13 × 10^−6^
	16	13,116,847	14,137,101	55	13,629,940^a^	13,011,210	0.446	−0.19 (0.06)	0.487	0.18 (0.05)	3.41 × 10^−6^
	18	32,88,714	4,302,911	3	3,802,911^a^	3,762,701	0.015	1.15 (0.24)	0.026	−0.27 (0.17)	1.64 × 10^−6^
	22	59,87,150	6,987,507	3	6,487,150^a^	6,411,683	0.062	0.23 (0.11)	0.040	−0.63 (0.14)	1.57 × 10^−6^
	29	46,651,577	47,656,859	2	47,156,859^a^	46,499,562	0.027	−0.69 (0.17)	0.032	0.37 (0.15)	2.27 × 10^−6^

^1^DHQ, development of hind quarter; DIT, development of inner thigh; DL, development of loin; TW, thigh width; WOW, width of withers.

^a^Intergenic variant.

^b^Intron variant.

^c^Upstream gene variant.

*SNP has not been mapped on the latest build.

**Table 11.** The location of the most significantly dimorphic SNPs, limited to the top 5, which were associated with the skeletal traits in the SI

							Male		Female		
Trait^1^	Chr	Start	End	No. of dimorphic SNPs	Most significantly dimorphic SNP	Updated position of most significantly dimorphic SNP (ARS-UCD 1.2)	Allele frequency	Allele effect (SE)	Allele frequency	Allele effect (SE)	Significance of dimorphism
WH	10	39,416,014	40,523,072	17	39,916,014^b^	39,827,207	0.003	−0.86 (0.32)	0.004	1.05 (0.29)	8.11 × 10^−6^
	15	79,224,399	80,224,427	2	79,724,399^d^	78,498,073	0.031	−0.23 (0.09)	0.016	0.55 (0.15)	9.47 × 10^−6^
	21	43,816,205	44,823,203	2	44,316,205^a^	43,898,184	0.005	−0.82 (0.25)	0.005	0.94 (0.27)	1.70 × 10^−6^
	23	29,940,021	31,111,779	72	30,451,606^a^	30,700,499	0.314	−0.14 (0.04)	0.319	0.13 (0.04)	9.09 × 10^−7^
	X	144,910,600	145,867,274	8	145,410,600^b^	134,272,382	0.007	−0.26 (0.19)	0.011	0.30 (0.22)	3.54 × 10^−8^
BL	1	68,029,603	69,086,059	16	68,580,516^bd^	67,982,646	0.476	0.11 (0.04)	0.483	−0.15 (0.04)	7.57 × 10^−7^
	6	12,731,620	13,787,338	4	13,287,338^a^	*	0.004	1.11 (0.26)	0.009	−0.44 (0.18)	1.01 × 10^−6^
	9	84,630,969	85,633,807	2	85,130,969^b^	84,014,933	0.025	−0.52 (0.12)	0.018	0.40 (0.14)	6.71 × 10^−7^
	26	23,562,542	24,623,399	7	24,110,489^a^	23,866,480	0.086	−0.20 (0.06)	0.059	0.30 (0.08)	6.31 × 10^−7^
	28	39,092,226	40,119,176	5	39,592,226^a^	39,277,437	0.348	−0.20 (0.04)	0.347	0.08 (0.04)	5.94 × 10^−7^
HW	7	2,278,460	3,359,162	15	27,85,141^a^	2,871,594	0.109	0.16 (0.05)	0.071	−0.35 (0.08)	2.81 × 10^−7^
	7	92,446,667	93,472,476	2	92,972,476^a^	90,587,933	0.029	−0.25 (0.10)	0.020	0.67 (0.15)	1.94 × 10^−7^
	8	23,629,760	24,686,981	6	24,129,760^c^	24,165,949	0.004	−0.84 (0.27)	0.003	1.66 (0.39)	1.51 × 10^−7^
	21	3,783,435	4,810,305	6	4,308,308^a^	4,167,947	0.129	−0.23 (0.05)	0.125	0.17 (0.06)	5.70 × 10^−7^
	X	138,125,669	139,135,827	3	138,625,669^a^	138,267,978	0.067	0.02 (0.06)	0.060	0.29 (0.10)	5.57 × 10^−7^
CW	2	116,794,370	119,288,912	3	117,931,744	*	0.009	0.31 (0.18)	0.005	−1.24 (0.26)	5.88 × 10^−7^
	6	35,397,549	36,711,379	11	36,185,495^b^	34,752,887	0.439	0.14 (0.03)	0.472	−0.13 (0.04)	2.20 × 10^−7^
	12	53,187,285	54,432,359	5	53,687,285^a^	53,340,630	0.208	0.07 (0.04)	0.228	−0.21 (0.05)	3.91 × 10^−6^
	16	57,116,725	58,168,280	26	57,657,852^b^	56,195,234	0.007	−0.82 (0.19)	0.011	0.43 (0.18)	1.85 × 10^−6^
	22	23,885,582	25,123,955	9	24,451,172^a^	24,349,773	0.086	0.18 (0.06)	0.121	−0.21 (0.06)	2.41 × 10^−6^
CD	1	80,65,635	9,123,471	237	88,582,433^a^	87,956,643	0.004	0.08 (0.24)	0.002	−2.64 (0.42)	1.98 × 10^−8^
	6	117,081,578	119,074,538	2	117,581,578^a^	112,778,288	0.008	0.54 (0.17)	0.003	−1.47 (0.34)	1.95 × 10^−7^
	7	90,595,916	91,626,272	3	91,121,080^a^	88,762,617	0.004	0.54 (0.25)	0.002	−2.06 (0.42)	1.27 × 10^−7^
	11	56,218,949	59,206,326	7	58,002,539^a^	58,081,253	0.002	0.36 (0.38)	0.002	−3.48 (0.60)	6.15 × 10^−8^
	12	5,835,154	6,893,124	2	63,35,154^a^	6,351,298	0.004	−0.07 (0.24)	0.002	−3.48 (0.60)	1.12 × 10^−7^

^1^WH, wither height; BL, back length; HW, hip width; CW, chest width; CD, chest depth.

^a^Intergenic variant.

^b^Intron variant.

^c^Downstream gene variant.

^d^Upstream gene variant.

*SNP has not been mapped on the latest build.

Few 1-kb windows containing a suggestive SNP overlapped among the muscular and skeletal traits. A single 1-kb window on BTA2, approximately 0.1 Mb from the *IWS1* gene, contained suggestively dimorphic SNPs for all of DIT, DL, and CW. Of the skeletal traits, no genomic windows exhibited suggestive associated dimorphism (Supplementary Figure S1e), and no window was suggestively associated with three or more muscular traits (Supplementary Figure S2e). Three 1-kb windows, all located on BTA18 between 3.78 and 3.80 Mb, contained dimorphic SNPs for both TW and WOW (Supplementary Figure S2e); these windows were located approximately 0.3 Mb from the *CNTNAP4* gene. One window located on BTA28 at 41.045 Mb, and six windows on the X chromosome between 114.572 and 114.578 Mb were dimorphic for both TW and DL. A single 1-kb window was common to each of DIT and TW (13.541 Mb on BTA8), DHQ and WOW (102.906 Mb on BTA6), and WH and BL (145.410 Mb on BTAX).

### Across breed

The numerically smaller breeds of AA, HE, and SI had approximately 1.5 times more SNPs segregating in only one sex than the numerically larger breeds of CH and LM. Of the total autosomal SNPs that were segregating in both sexes, between 86% (AA) and 94% (LM) had the same minor alleles in both sexes. However, differences (*P* ≤ 0.05) in allele frequencies between the sexes were observed for between 36% (LM) and 66% (AA) of the total autosomal SNPs that were segregating in both sexes.

The vast majority of SNPs and 1-kb windows associated with any one trait were breed specific. The most windows displaying dimorphic characteristics in common for more than one breed was for CW in both the CH and LM with nine 1-kb windows common to these breeds occurring on BTA2 at 89.527 Mb (*n* = 1), on BTA12 between 87.128 and 87.303 Mb and containing the *ENSBTAG00000032038* gene (*n* = 6), and on BTA24 between 31.661 and 31.671 Mb (*n* = 2). Also for CW, a single 1-kb window on BTA6 approximately 0.1 Mb from the *SLC34A2* gene displayed dimorphic associations in both the AA and the CH. A single 1-kb window containing the *ENSBTAG00000046311* gene on BTA10 had dimorphic associations with HW in both the AA and SI. In the CH and the SI, two common windows, one at 91.126 Mb on BTA7 and one at 70.827 Mb on BTA9, exhibited dimorphism with CD in both breeds.

## Discussion

Although several studies in cattle have investigated the presence of sexual dimorphism using a quantitative genetics approach (Crews and Kemp, 2001; van der Heide et al., 2016; Bittante et al., 2018; Doyle et al., 2018), no previous study in cattle has attempted to detect evidence of sexual dimorphism at the genome level or to compare these effects across multiple breeds of cattle. From previous quantitative genetics studies, differences in genetic parameters by cattle sex have been observed for growth rate (Koch and Clark, 1955; Marlowe and Gaines, 1958), postweaning gain (van der Heide et al., 2016), feed intake and efficiency, fleshiness scores, carcass weight and yield (Bittante et al., 2018), as well as longissimus muscle area and backfat (Crews and Kemp, 2001). In contrast, negligible differences in genetic parameters between the sexes were detected for early growth traits such as weaning weight (Koch and Clark, 1955; van der Heide et al., 2016).

A single trait measured in different environments (or different sexes) can be regarded as separate traits which are genetically correlated (Falconer, 1952). In many situations, the genetic correlations of the same trait taken in different environments are less than unity, indicating that selection occurring in one environment may not be optimal for performance in the other environment (Mulder and Bijma, 2005). This represents a genotype-by-environment interaction although Robertson (1959) postulated that the genetic correlation between environments would need to be weaker than 0.80 to be considered of importance for breeding purposes. In quantitative genetics studies on dimorphism, it is not necessarily the environment that is causing the genetic correlations to differ from unity, but possibly the effect of dimorphism (which is often cofounded with environment). Weaker than unity genetic correlations between the sexes may be indicative of many factors including the alleles having a different substitution effect in each sex; these differences may be due to intersex differences in effect sizes or the sign of the allele substitution effect differing by sex.

Bittante et al. (2018), while investigating the effects of sexual dimorphism on the fattening performance and muscling of young Belgian Blue and Piedmontese dairy cross bulls and heifers, noticed that the effects of dimorphism were greater in the Belgian Blues than the Piedmontese suggesting that the effects of sexual dimorphism may actually differ by breed. Because linear-type traits which describe the skeletal and muscular conformation of an animal are related to many performance traits such as animal live weight (Mc Hugh et al., 2012), carcass merit (Mukai et al., 1995; Conroy et al., 2010), primal cut yields (Berry et al., 2019), and feed intake (Veerkamp and Brotherstone, 1997; Crowley et al., 2011), it is plausible that the underlying variome of animals contributing to differences in their skeletal or muscular characteristics may also exhibit sexual dimorphism; it is also plausible that these regions exhibiting dimorphism may differ by breed. The data set used in the present study was particularly useful to test this hypothesis in that all linear-type traits were assessed in all breeds and sexes using the same scale by the same classifiers and, therefore, a direct comparison of sex effects as well as commonalities of detected regions across breeds was possible.

Using a data set of 32,725 males and 30,887 females of the CH and LM breeds, Doyle et al. (2018) estimated variance components for 18 type traits in both sexes separately; the type traits included in the present study were those represented in that study and included functional, skeletal, and muscular subjective measures. Numerical differences in variance estimates were detected between both sexes in each breed while intersex differences in heritability estimates were only significant (*P* < 0.05) for BL, WH, and DHQ in the CH, with no differences observed in the LM (Doyle et al., 2018). Within trait genetic correlations between each of the 18 type traits in each sex were all stronger than 0.90 (Doyle et al., 2018); because Robertson (1959) concluded that genetic correlations had to be weaker than 0.80 to be impactful, Doyle et al. (2018) concluded a lack of dimorphism in their study. Nonetheless, genetic correlations are derived from the entire genome and therefore may not capture the granularity achieved by investigation of specific regions of the genome, as undertaken in the present study. This is especially true when only a few regions exhibit dimorphism and the extent of dimorphism in these regions may be small. Indeed the results from the present study indicate that, in fact, only a few regions exhibit dimorphism and the effects are small. Moreover, although the analyses conducted in the present study only considered SNPs segregating in both sexes, the existence of SNPs that were monomorphic in one sex may also be indicative of dimorphism.

### The X chromosome

The X chromosome is the second largest chromosome in the bovine genome and accounts for over 6% of the total physical genome (148,823,899 bp; Zimin et al., 2009); it is, however, regularly discarded from genome-based studies in cattle due the inheritance of the X chromosome being different to the autosomes. Males are heterogametic (XY) and the females are homogametic (XX; Fernando and Grossman, 1990); therefore, male offspring inherit their X chromosome from their dam only, while female offspring inherit one copy of the X chromosome from their dam and the other from their sire. Furthermore, a small region of the X chromosome, known as the PAR, is homologous to the Y chromosome and is inherited like an autosome with some recombination occurring during meiosis (Van Laere, et al., 2008; Su et al., 2014).

Ignoring the X chromosome could lead to important biological functions being missed and could also impact the accuracy of genomic evaluations (Lyons et al., 2014; Su et al., 2014; Mao et al., 2016). A previous study on the role of the sex chromosomes in dimorphism (Rice, 1984) revealed that while sex chromosomes were not required for the evolution of sexual dimorphism, they facilitated the evolution of sexual dimorphism for a wider range of traits than would have occurred without them and that X-linked genes in particular had a large role in the evolution of sexually dimorphic traits. In the present study, only 406 SNPs located on the X chromosome expressed sexual dimorphism in at least one breed or trait. The low number of dimorphic SNPs on the X chromosome may be a function of possible allelic content variation where females have two copies of an allele and males only have one, thus interfering with the detection of dimorphic SNPs on the X chromosome. However, previous studies have discovered that in most cases, sex chromosomes are only required to initiate sexual dimorphism and the corresponding genes are mostly located on the autosomes (Saifl and Chandra, 1999; Fairbairn and Roff, 2006).

Previous studies have linked mutations on the X chromosome to andrological and growth traits in beef cattle (Lyons et al., 2014) as well as the length of productive life in dairy cattle (Saowaphak et al., 2017). In the present study, all sexually dimorphic SNPs located on the X chromosome for any trait in any of the breeds had a greater effect size in females than in males; this is in agreement with a previous study on sexual dimorphic gene expression in cattle using RNA-seq which stated that all X-chromosomal sexually dimorphic genes had a greater effect in females than males (Seo et al., 2016). In other species, such as *Drosophila melanogaster*, it has been proven that an X-linked recessive mutation that benefits males will accumulate faster as expression in males is hemizygous and there will be no masking by dominance (Gibson et al., 2002); however, the male-biased expression of these alleles reduces as the allele becomes more frequent in the population which enables counter-selection in the females to halt the spread of this male-biased allele.

### Allele frequencies

The number of SNPs included in each of the analyses in the present study differed by both sex and breed due solely to the SNP not always segregating in each sub-population. Although the vast majority of SNPs in the present study had the same minor allele in both sexes, differences between sexes in allele frequencies still exist. In humans, it has been hypothesized that intersex differences in allele frequencies occur during the initial evolution of sexual dimorphism due to males and females having different fitness optima for a phenotype (Rice, 1984; Lucotte et al., 2016). In cattle, differences in allele frequencies between the sexes may also have arisen due to these differential fitness optima for males and females where selection occurred at a given locus in one sex but not the other or due to different selection pressures at that locus in each sex (Lucotte et al., 2016); for example, selection for a female trait could have very different effects on genetically correlated traits in females compared with those traits in males (Bittante et al., 2018).

Intersex differences in the frequency of a given allele may also be due to intersex differences in recombination rates; up to 75% of species that undergo recombination in their genome have different recombination rates per sex (Burt et al., 1991; Wyman and Wyman, 2013). In the majority of species, the male recombination rate is generally lower than in females (Poissant et al., 2010; Wyman and Wyman, 2013). It is thought that this lower recombination rate is advantageous to males as it maintains combinations of beneficial genes that have undergone sexual selection (Trivers, 1988); however, studies in both cattle (Ma et al., 2015) and sheep (Maddox and Cockett, 2007) reported that the male recombination rate in these species is actually higher than the females.

### Across breed

Based on a series of within-breed genome-wide associations undertaken across both sexes combined using the data from the present study, Doyle et al. (2020a, b) detected little to no commonality in the genomic regions associated with each type trait across breeds. This indicates that the underlying genetic basis of the same trait in each breed is quite different; therefore, it was somewhat expected that the regions exhibiting dimorphism may also differ by breed. In general, the British breeds (AA and HE) had fewer suggestively or significantly dimorphic SNPs than the continental breeds but the British breeds had a greater percentage of SNPs exhibiting significant differences in allele frequencies between the sexes than the continental breeds. The location of the most significantly dimorphic SNPs also differed across the breeds. The differences observed between the breeds may be due to actual differences in the genetic basis of sexual dimorphism among the breeds, as previously observed between the Belgian Blue and Piemontese cattle breeds (Bittante et al., 2018), or may be simply due to differences in the statistical power to detect QTL due to the differences in breed-specific population sizes (Doyle et al., 2020a). Actual differences in the genetics underlying each trait may be attributable to different mutations affecting specific genes in each breed, such as the breed-specific *MSTN* mutations, or may, more likely be attributable to different QTL being affected by different selection pressures within each breed (Bittante et al., 2018).

### Genes exhibiting dimorphism

SNPs exhibiting sexually dimorphism were located within or close to a number of different genes that have previously been associated with muscularity and/or size in cattle (Doyle et al., 2020a, b). Three genes on BTA2 (*NAB1*, *COL5A2*, and *IWS1*) containing dimorphic SNPs associated with multiple traits are thought to either be in strong linkage disequilibrium with *MSTN* (Grade et al., 2009) or have been previously identified as being located within a QTL also containing *MSTN* that was associated with muscularity in beef cattle (Doyle et al., 2020a). The *MSTN* gene has already been documented as being responsible for muscular hypertrophy in cattle (Grobet et al., 1997; McPherron and Lee, 1997) and is widely known as the causal variant for many muscularity and carcass traits in cattle (Casas et al., 2000; Allais et al., 2010; Purfield et al., 2019). Another candidate gene for muscularity that exhibited evidence of sexual dimorphism was the *PDHX* gene on BTA15 that contained dimorphic SNPs for 3 of the muscularity traits in CH and has previously been associated with carcass quality traits in beef cattle (Karisa et al., 2013).

## Conclusion

Although many significantly and suggestively sexually dimorphic SNPs associated with the muscular and skeletal type traits were identified in the present study, the location and effect sizes of these tended to be both trait specific and breed specific. Both the allele substitution effect sizes and the allele frequencies of the dimorphic SNPs also differed by sex. This indicates that although sexual dimorphism exists in cattle at a genome level, it occurs at a low frequency but also differs both by trait and by breed.

## Supplementary Material

skab070_suppl_Supplementary_Figures_S1_S2

skab070_suppl_Supplementary_Table_S1

skab070_suppl_Supplementary_Table_S2

## Abbreviations

BTA: *Bos Taurus* autosome
GRM: genomic relationship matrix
HD: high density
MAF: minor allele frequency
PAR: pseudoautosomal region
QTL: quantitative trait loci
SNP: single-nucleotide polymorphism
WGS: whole-genome sequence
