# Supplementary material for: Identification of genomic regions that exhibit sexual dimorphism for size and muscularity in cattle
Source: J Anim Sci. 2021 Mar 2;99(5):skab070. doi: 10.1093/jas/skab070 (PMC8480176; doi:10.1093/jas/skab070)
Supplement: skab070_suppl_Supplementary_Figures_S1_S2 [file skab070_suppl_supplementary_figures_s1_s2.docx]

| **a)** | **b)** |
| --- | --- |
| **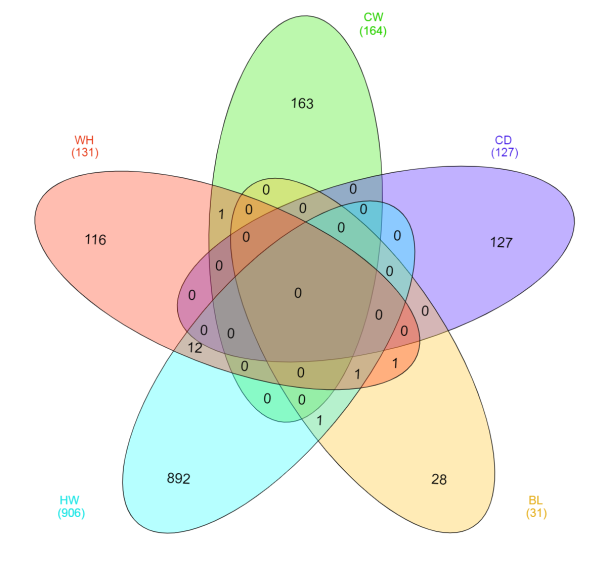** | **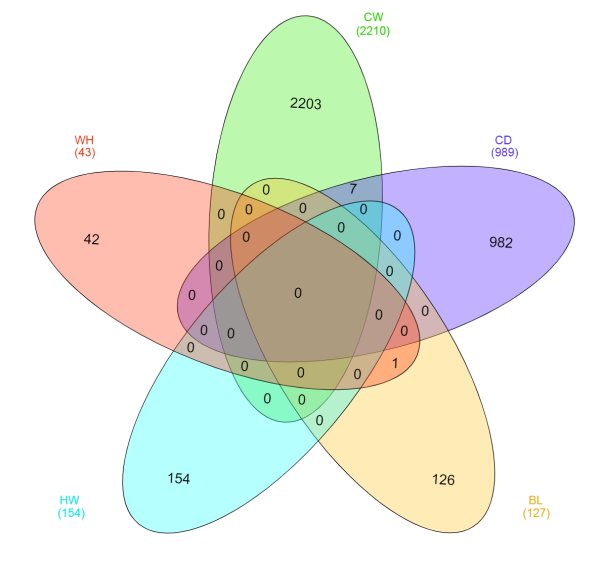** |
| **c)** | **d)** |
| **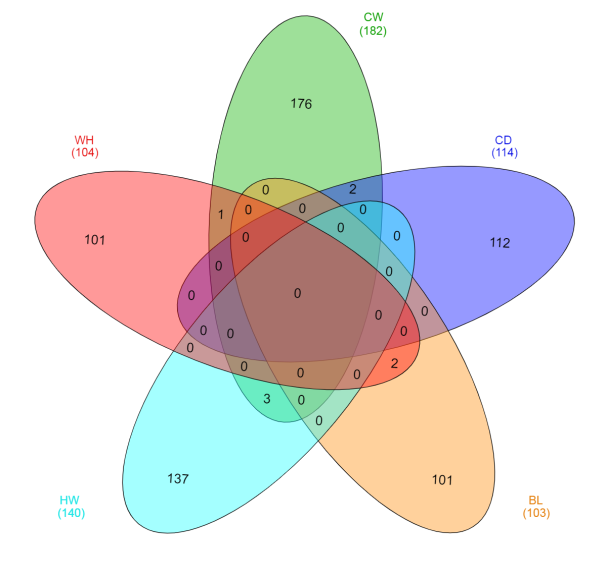** | **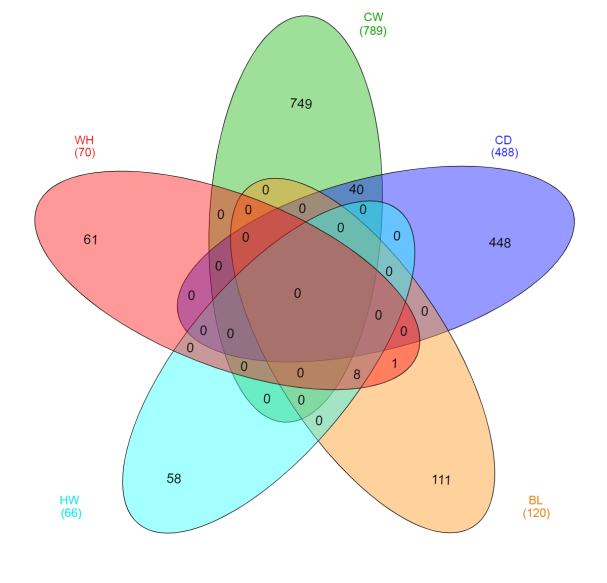** |
| **e)** |  |
| 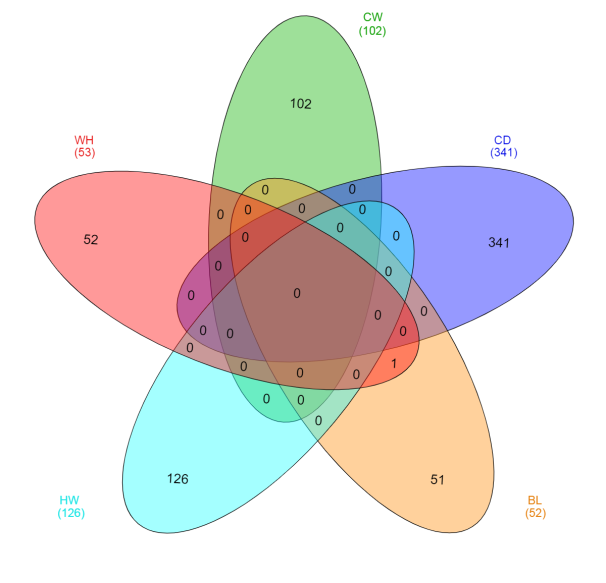 |  |

Figure S1: Overlapping 1kb regions that contain at least one suggestively or significantly dimorphic SNP for the 5 skeletal traits in a) Angus, b) Charolais, c) Hereford, d) Limousin, and e) Simmental

*trait abbreviations: CW = chest width, CD = chest depth, BL = back length, HW = hip width, WH = wither height

| **a)** | **b)** |
| --- | --- |
| **a)** | **b)** |
| **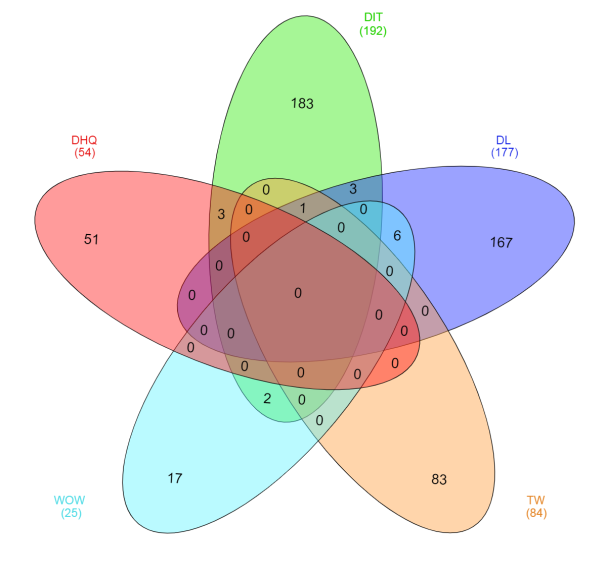** | **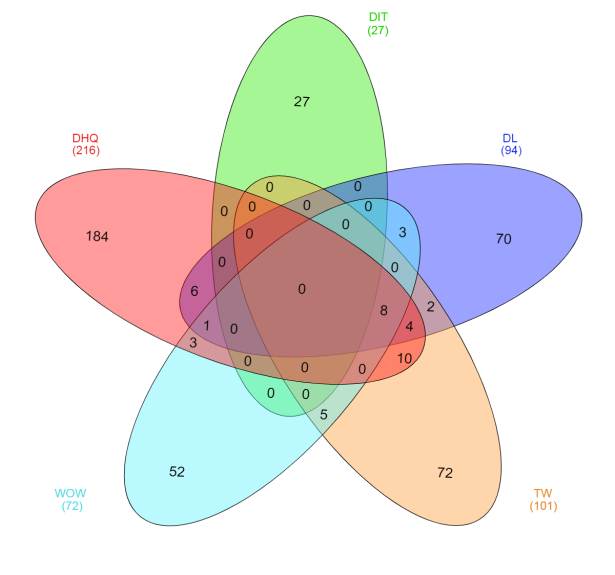** |
| **c)** | **d)** |
| **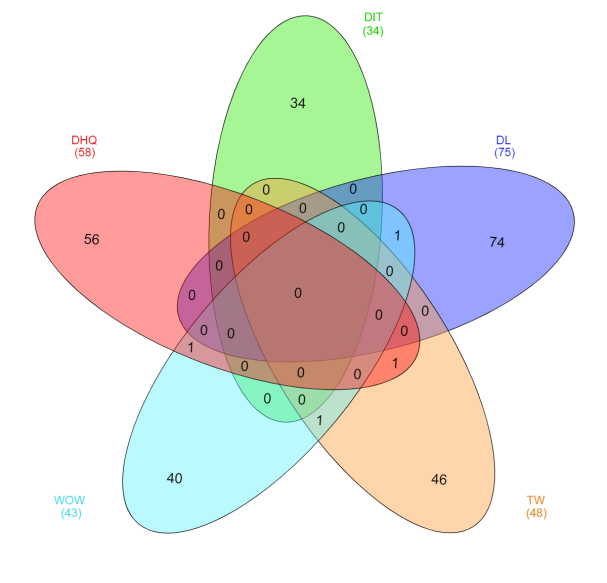** | **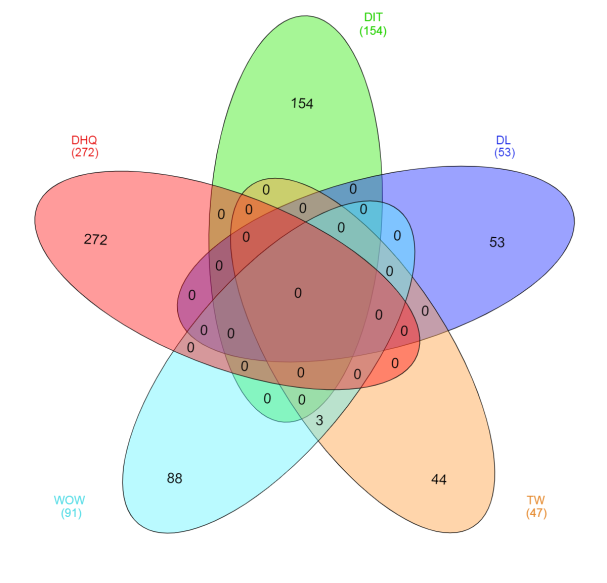** |
| **e)** |  |
| 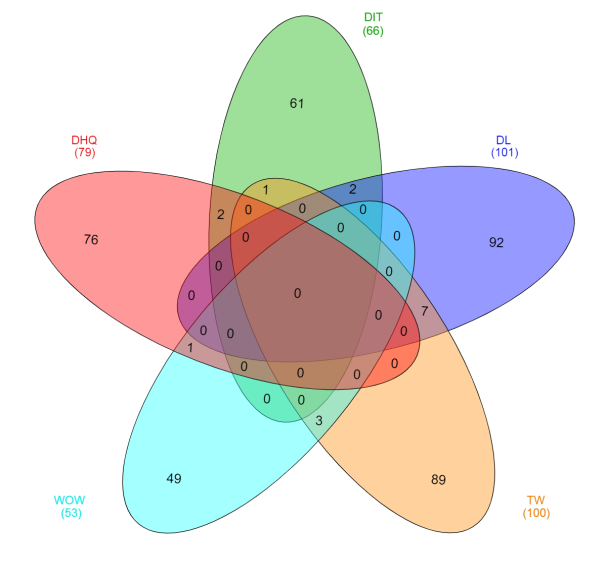 |  |

Figure S2: Overlapping 1kb regions that contain at least one suggestively or significantly dimorphic SNP for the 5 muscular traits in a) Angus, b) Charolais, c) Hereford, d) Limousin and e) Simmental

*trait abbreviations: DHQ = development of hind quarter, DIT = development of inner thigh, DL = development of loin, TW = thigh width, WOW = width of wither.
