# Supplementary material for: Identification of genomic regions that exhibit sexual dimorphism for size and muscularity in cattle
Source: J Anim Sci. 2021 Mar 2;99(5):skab070. doi: 10.1093/jas/skab070 (PMC8480176; doi:10.1093/jas/skab070)
Supplement: skab070_suppl_Supplementary_Table_S1 [file skab070_suppl_supplementary_table_s1.docx]

Table S1: The mean (µ) and phenotypic standard deviation (SD) of each linear type trait by breed and sex.

|  |  | Angus | | | | |  | Charolais | | | | |  | Hereford | | | | |  | Limousin | | | | |  | Simmental | | | | |
| --- | --- | --- | --- | --- | --- | --- | --- | --- | --- | --- | --- | --- | --- | --- | --- | --- | --- | --- | --- | --- | --- | --- | --- | --- | --- | --- | --- | --- | --- | --- |
|  |  | Male | |  | Female | |  | Male | |  | Female | |  | Male | |  | Female | |  | Male | |  | Female | |  | Male | |  | Female | |
| Traits | Scale | µ | SD |  | µ | SD |  | µ | SD |  | µ | SD |  | µ | SD |  | µ | SD |  | µ | SD |  | µ | SD |  | µ | SD |  | µ | SD |
| *Muscular* | *1 to 15* |  |  |  |  |  |  |  |  |  |  |  |  |  |  |  |  |  |  |  |  |  |  |  |  |  |  |  |  |  |
| Development of hind quarter | low - high | 7.96 | 1.12 |  | 7.07 | 1.44 |  | 9.83 | 1.28 |  | 8.57 | 1.22 |  | 7.64 | 1.24 |  | 6.82 | 1.36 |  | 10.38 | 1.24 |  | 9.34 | 1.26 |  | 9.90 | 1.21 |  | 8.55 | 1.23 |
| Development of loin | low - high | 8.19 | 1.24 |  | 7.37 | 1.30 |  | 10.00 | 1.42 |  | 8.58 | 1.27 |  | 8.21 | 1.24 |  | 7.39 | 1.11 |  | 10.00 | 1.39 |  | 8.87 | 1.31 |  | 9.86 | 1.39 |  | 8.56 | 1.40 |
| Thigh width | narrow - wide | 7.86 | 1.28 |  | 6.99 | 1.41 |  | 9.68 | 1.51 |  | 8.34 | 1.30 |  | 7.71 | 1.36 |  | 6.78 | 1.20 |  | 9.62 | 1.56 |  | 8.60 | 1.52 |  | 9.69 | 1.45 |  | 8.35 | 1.38 |
| Development of inner thigh | low - high | 7.71 | 1.33 |  | 6.85 | 1.50 |  | 9.57 | 1.49 |  | 8.32 | 1.28 |  | 7.37 | 1.49 |  | 6.53 | 1.40 |  | 10.09 | 1.39 |  | 8.92 | 1.32 |  | 9.64 | 1.27 |  | 8.20 | 1.28 |
| Wither width | narrow - wide | 8.29 | 1.51 |  | 7.40 | 1.52 |  | 9.92 | 1.55 |  | 8.53 | 1.38 |  | 8.06 | 1.49 |  | 7.31 | 1.20 |  | 10.00 | 1.49 |  | 8.85 | 1.36 |  | 9.78 | 1.56 |  | 8.35 | 1.62 |
| *Skeletal* | *1 to 10* |  |  |  |  |  |  |  |  |  |  |  |  |  |  |  |  |  |  |  |  |  |  |  |  |  |  |  |  |  |
| Chest width | narrow - wide | 5.78 | 0.92 |  | 5.22 | 0.94 |  | 6.35 | 0.97 |  | 5.76 | 0.85 |  | 5.78 | 0.89 |  | 5.39 | 0.80 |  | 5.97 | 0.95 |  | 5.48 | 0.93 |  | 6.33 | 0.90 |  | 5.70 | 0.90 |
| Chest depth | shallow - deep | 6.45 | 0.88 |  | 6.09 | 0.88 |  | 6.93 | 0.91 |  | 6.34 | 0.85 |  | 6.50 | 0.87 |  | 6.10 | 0.82 |  | 6.59 | 0.90 |  | 6.12 | 0.86 |  | 7.08 | 0.86 |  | 6.53 | 0.78 |
| Wither height | small- tall | 5.55 | 0.97 |  | 5.08 | 1.02 |  | 6.10 | 1.14 |  | 5.66 | 1.05 |  | 5.64 | 1.01 |  | 5.16 | 0.98 |  | 5.89 | 1.08 |  | 5.45 | 1.06 |  | 6.49 | 1.00 |  | 5.82 | 0.97 |
| Back length | short - long | 6.42 | 0.98 |  | 5.88 | 1.04 |  | 6.88 | 1.07 |  | 6.41 | 0.99 |  | 6.47 | 1.04 |  | 6.00 | 0.97 |  | 6.72 | 1.06 |  | 6.34 | 1.06 |  | 7.28 | 0.92 |  | 6.73 | 0.93 |
| Hip width | narrow - wide | 5.48 | 0.91 |  | 5.40 | 1.06 |  | 5.80 | 0.95 |  | 5.51 | 0.94 |  | 5.65 | 0.87 |  | 5.68 | 0.91 |  | 5.71 | 1.04 |  | 5.46 | 1.07 |  | 6.10 | 0.90 |  | 5.76 | 0.98 |
