# Supplementary material for: Identification of genomic regions that exhibit sexual dimorphism for size and muscularity in cattle
Source: J Anim Sci. 2021 Mar 2;99(5):skab070. doi: 10.1093/jas/skab070 (PMC8480176; doi:10.1093/jas/skab070)
Supplement: skab070_suppl_Supplementary_Table_S2 [file skab070_suppl_supplementary_table_s2.docx]

Table S2: The number of phenotyped and genotyped animals by sex and breed and along with the number of SNPs removed during quality control and included in the final analysis.

|  | Angus | |  | Charolais | |  | Hereford | |  | Limousin | |  | Simmental | |
| --- | --- | --- | --- | --- | --- | --- | --- | --- | --- | --- | --- | --- | --- | --- |
|  | Male | Female |  | Male | Female |  | Male | Female |  | Male | Female |  | Male | Female |
| Phenotyped animals | 1,812 | 1,544 |  | 16,145 | 14,904 |  | 1,582 | 1,422 |  | 17,930 | 17,229 |  | 4,489 | 4,143 |
| Genotyped animals | 1,044 | 400 |  | 4,641 | 1,792 |  | 727 | 402 |  | 5,772 | 2,973 |  | 956 | 742 |
| Sequence SNPs | 42,920,277 | 42,920,277 |  | 42,920,277 | 42,920,277 |  | 42,920,277 | 42,920,277 |  | 42,920,277 | 42,920,277 |  | 42,920,277 | 42,920,277 |
| SNPs removed during minor allele frequency edit | 25,815,321 | 26,967,481 |  | 24,288,291 | 24,898,964 |  | 25,107,580 | 25,862,892 |  | 24,082,484 | 24,528,104 |  | 23,970,492 | 24,525,773 |
| Removed due to poor imputation accuracy | 563,043 | 550,636 |  | 577,712 | 572,365 |  | 571,545 | 562,481 |  | 780,880 | 624,936 |  | 692,610 | 580,207 |
| SNPs included in analysis | 16,541,913 | 15,402,160 |  | 18,054,274 | 17,448,948 |  | 17,241,152 | 16,494,904 |  | 18,056,913 | 17,767,237 |  | 18,257,175 | 17,814,297 |
| Total number of SNPs present in both sexes | 15,008,408 | |  | 17,227,625 | |  | 15,991,751 | |  | 17,482,131 | |  | 17,319,250 | |
